# Forensic autopsies in Norway 1996–2017: A retrospective study of factors associated with deaths undergoing forensic autopsy

**DOI:** 10.1177/1403494821997208

**Published:** 2021-03-08

**Authors:** Christian Lycke Ellingsen, G. Cecilie Alfsen, Geir Sverre Braut

**Affiliations:** 1 Department of Pathology, Stavanger University Hospital, Norway; 2 Department of Global Public Health and Primary Care, University of Bergen, Norway; 3 Department of Pathology, Akershus University Hospital, Norway; 4 Faculty of Medicine, University of Oslo, Norway; 5 Department of Research, Stavanger University Hospital, Norway

**Keywords:** Forensic autopsy, cause of death statistics

## Abstract

**Aims:**

Forensic autopsies are important for the investigation of deaths with a legal or public-health interest, as well as being a source for cause-of-death statistics. The aim of this study was to investigate the use of forensic autopsies in Norway, with a special emphasis on geographical variation.

**Methods:**

Data from the Norwegian Cause of Death Registry for the years 1996–2017 included 920,232 deaths and 37,398 forensic autopsies. We used logistic regression to identify factors that were associated with the proportion of forensic autopsies, grouped according to the registered cause of death. Explanatory variables were age and sex, place of death, police district, population size and urbanity level of the municipality and distance to the autopsy facility.

**Results:**

The proportion of deaths undergoing forensic autopsy was 4.1%, with the highest being homicides (96.6%) and the lowest being deaths from natural causes (1.7%). Variation between police districts was 0.9–7.8%, and the span persisted during the study period. The most important explanatory variables across the strata were place of death (there were few autopsies of deaths in health-care facilities), police district and age of the deceased. Distance to the autopsy facility, sex, population size and the level of urbanity had only a minor influence. The variation between police districts was not fully accounted for by the other investigated factors.

**Conclusions:**

**Unjustified differences in the frequency of autopsies may lead to insufficient investigation of possible unnatural deaths. In worst-case scenarios, homicides or other criminal cases might remain undetected. It may also introduce spurious shifts in the cause-of-death statistics.**

## Introduction

A forensic autopsy is part of the investigation of a death that to some degree is of public interest. The most important function is to investigate a possible criminal cause of death. Different states and countries have different death investigation systems, but they all aim to cover outright homicides and deaths that might be disguised criminal cases. Many jurisdictions include deaths where the suspicion of homicide is low but where there is a public interest in investigating or documenting the cause of death. Among these are deaths caused by recklessness or negligence, such as traffic accidents, workplace accidents or medical misadventure, or deaths that have important public-health issues, such as suicides or deaths related to drug abuse [1].

As rules may vary between locations, the number of deaths eligible for a forensic autopsy also varies. The number of deaths that actually undergo a forensic autopsy also depends on compliance with the regulations.

In Norway, Igeltjørn and Nordrum [2] found that the proportion of forensic autopsies for road traffic accidents in two neighbouring counties varied from 49% to 70%. Frost et al. [3] found differences in the proportion of forensic autopsies between the same two counties according to age, sex and cause of death. For example, the proportion of autopsies for suicides varied from 11% to 91%. In Denmark, Winkel et al. [4] found that the proportion of forensic autopsies for sudden death in young people varied from 60% to 88% between regions. Finland has had one of the highest proportions of forensic autopsy in the world (23.8% in 2004), but even there, differences have been noticed in the proportion of autopsies between geographical regions, as well as a decreasing proportion as the age of the deceased increased [5]. In Austria, there was a lower proportion of non-forensic autopsies for people dying at home in regions distant from autopsy facilities [6].

In Norway, the police must be notified if a death has a possible non-natural cause [7–10]. This includes all injury deaths, as well as sudden and unexpected deaths, deaths in custody, medical misadventures and children dying outside health-care facilities. Based on the information received, the police decide whether to initiate an investigation and request a forensic autopsy [11,12].

According to The Norwegian Board of Forensic Medicine, the forensic autopsy rate varies between geographical regions in Norway [13], but no thorough analysis has yet been performed of factors that might influence the request of a forensic autopsy.

The aim of this study was to examine the use of forensic autopsies in Norway for the years 1996–2017. We sought to describe variations in the autopsy proportions in different geographical locations and causes of death, and to explore possible explanatory factors such as: sex, age, (type of) place of death, police district, the population size and level of urbanity of the municipality and distance to the autopsy facility.

## Methods

### Data materials

The Norwegian Cause of Death Registry (NCoDR) at the Norwegian Institute of Public Health [14] supplied data concerning all deaths among Norwegian residents for the years 1996–2017 (*N*=930,589). We chose to use 1996 as the start of the study period, as the information on autopsies is incomplete for earlier years. We used the following variables: calendar year of death, sex, age at death, underlying cause of death (ICD-10 code), the (type of) place of death, the municipality where the death took place, whether an autopsy (forensic or medical) was performed and the autopsy laboratory. Additional data on the number of forensic autopsies were collected from the Norwegian Board of Forensic Medicine [13] and the Norwegian Society of Pathology [15]. The categories for grouping the underlying cause of death and the (type of) place of death are shown in the Supplemental Tables.

We collected population data from Statistics Norway [16]. Each municipality is classified on a six-level population scale and a seven-level urbanity–rurality (centrality) index. This is a compound scale based on the distance to population centres and the size of these centres. We retrieved map data from the Norwegian Mapping Authority [17] and information about which municipalities are included in each police district from the National Police Directorate [18]. During the study period, there were some adjustments in the structure of municipalities and police districts in Norway. To ensure comparability, we recoded the geographical and population data to the structure as it was in 2012.

We calculated the distance by road from the centre of the municipality of death to the autopsy facility serving the police district using a web service at the Norwegian Public Roads Authority [19]. Due to some shifts in the autopsy facilities serving each police district, the distance to the facility performing the most autopsies from each municipality was used as a default for the entire period.

### Ethical approval

The project was approved by the Regional Committee for Medical and Health Research Ethics and in consultation with the Data Protection Officer at Stavanger University Hospital.

## Methods

We used multiple logistic regression to investigate factors that could influence the probability of a forensic autopsy being performed. We partitioned the data into eight groups by the registered underlying cause of death. Explanatory variables were: calendar year of death in three periods, sex, age at death in 10-year groups, (type of) place of death in five groups, police district (*N*=27), population of the municipality in six groups, centrality index (seven-level scale) and distance to autopsy facility in 50 km intervals. Since the effects were not linear across the levels, all factors were used as unordered categorical variables.

First, we investigated each independent variable alone (univariate) before we entered all variables into a multiple predictors model. We used R v 3.6.1 (R Foundation for Statistical Computing, Vienna, Austria) with additional packages from the tidyverse collection [20], sf [21] and logistf [22] for all analyses. For logistic regression, we calculated odds ratios with 95% confidence intervals, likelihood ratio statistics (–2LogLikelihood) and two-sided *p*-values, with <0.05 considered statistically significant. To avoid unstable estimates caused by separation, we used Firth’s penalised likelihood method [23]. Binomial uncertainty intervals were calculated by Wilson’s interval method.

## Results

### Deaths and forensic autopsies

For the years 1996–2017, there were 930,589 deaths registered in the NCoDR. The total number of forensic autopsies reported to the NCoDR was 37,404 (4.1% of all deaths). After exclusion of deaths abroad or outside mainland Norway and deaths lacking information on the municipality or cause of death, 920,232 total deaths and 37,398 forensic autopsies remained.

The proportion of deaths undergoing forensic autopsy has been reasonably stable, ranging between 3.7% and 4.5% during the study period. There was no significant trend (Cochran–Armitage test for trend, χ^2^=0.07, *p*=0.79). The forensic autopsy rate (the number of forensic autopsies per 100,000 people) declined from 44.5 in 1998 to 30.5 in 2017.

The proportion of forensic autopsies varies between different causes of death. Almost all (96.6%) registered homicides undergo forensic autopsy, whereas around two out of three (63.7%) of suicides, approximately half (52.7%) of traffic deaths and only a few accidental falls (5.2%; Table I) are subject to autopsy. Only 1.7% of deaths from natural causes undergo forensic autopsy. However, they still constitute the single largest group of the autopsies (14,341; 38% of all forensic autopsies).

**Table I.** Proportions of forensic autopsies according to different causes of death.

Cause of death	Autopsies	Deaths	Proportion undergoing forensic autopsy (%)	Percentage of all forensic autopsies (%)
1. Natural	14,341	830,410	1.7	38.3
2. Ill-defined	889	30,082	3.0	2.4
3. Traffic accidents	2946	5632	52.3	7.9
4. Accidental falls	1050	20,307	5.2	2.8
5. Accidental poisonings	6090	7719	78.9	16.3
6. Other external causes of death	3602	9097	39.6	9.6
7. Suicide	7642	11,992	63.7	20.4
8. Homicide	844	874	96.6	2.3
Missing cause of death	0	4401	0	0

Data from the Norwegian Cause of Death Registry, 1996–2017.

### Age and sex

The median age of the deceased undergoing forensic autopsy was 50 years compared to 82 years for those not autopsied. In the age group 20–29 years, 59.5% of the deceased underwent forensic autopsy compared to 0.2% in the age group 90+. A total of 2.3% of deceased women and 6.0% of deceased men underwent forensic autopsy.

### (Type of) place of death

Very few deaths in health-care institutions (1.3% in hospitals and 0.1% in nursing homes) underwent forensic autopsy. The proportion was higher for those dying at home (12.9%) and highest for those dying at other locations (36.2% dying at other known location, 27.9% where the location was not specified).

### Police districts

**Figure 1.**
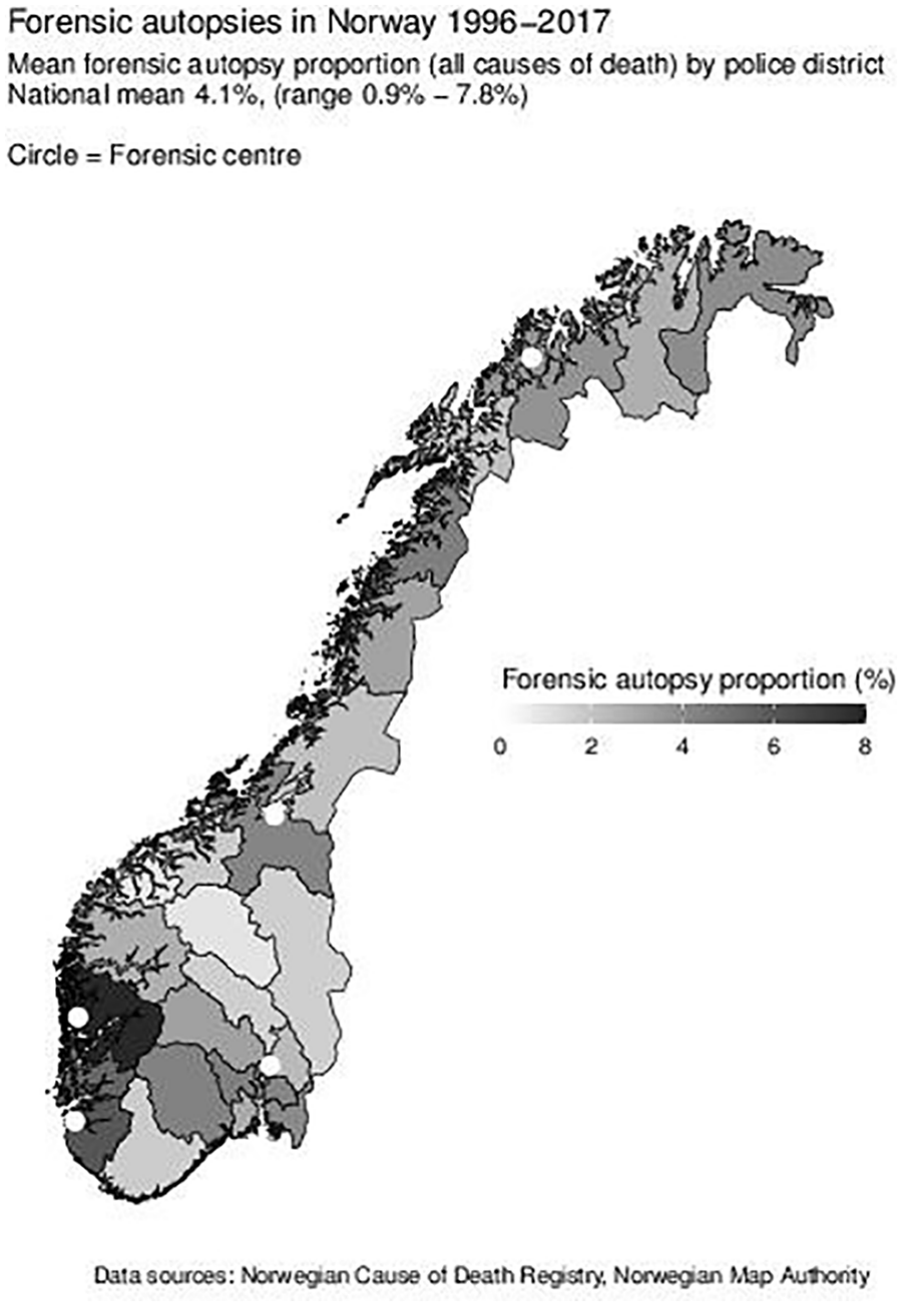
Proportion of forensic autopsies by police district.

**Figure 2.**
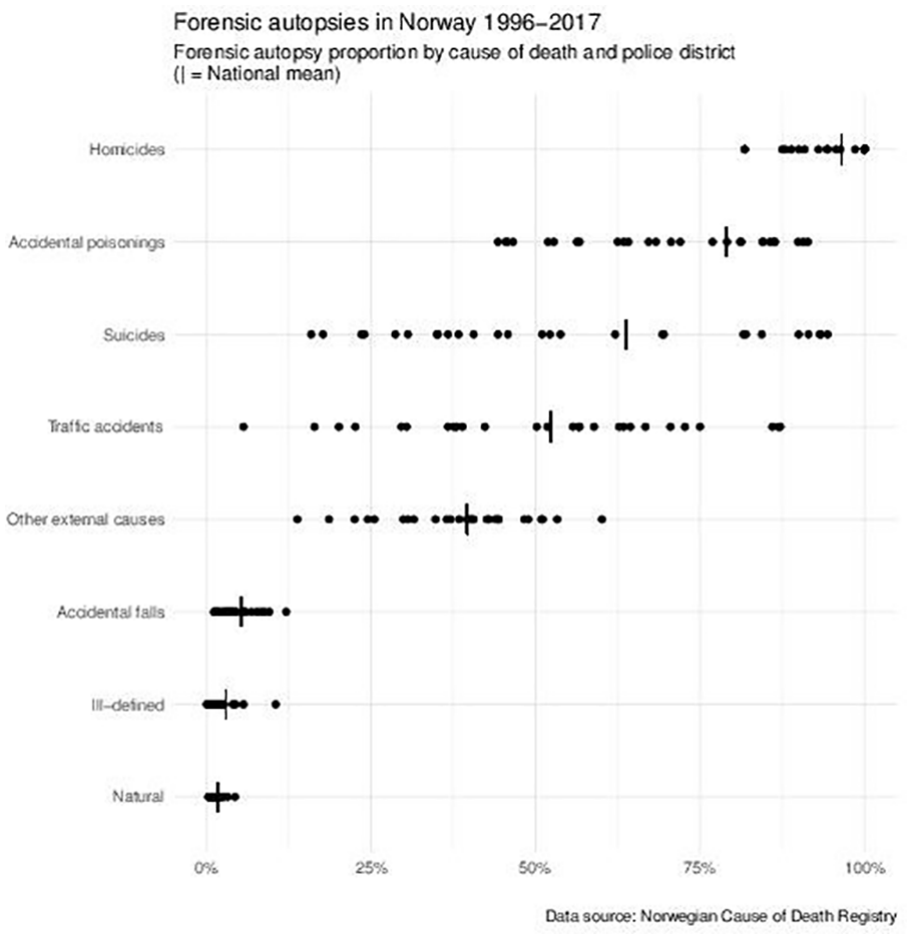
Proportion of forensic autopsies by cause of death and police district.

The proportion of forensic autopsies varies by a factor of almost nine from the police district with the highest proportion (Hordaland, 7.9%) to that with the lowest proportion (Gudbrandsdal, 0.9%; coefficient of variation (CV) 51%; Figure 1, map). The variation between police districts did not become smaller during the study period (Table II); the CV in both parts of the study period was 53%. Even if there were some changes in the autopsy proportion within each police district, no district changed rank from the highest to lowest third or vice versa. We also found a large variation between districts for the autopsy proportion for different causes of death. This was most pronounced for road traffic accidents and suicides (Figure 2).

**Table II.** Proportions of forensic autopsies according to police district and time periods.

	1996–2017				1996–2006 (First part of study period)				2007–2017 (Last part of study period)			
	All ages		0–59 years		All ages		0–59 years		All ages		0–59 years	
	% (95% CI)	Rank	% (95% CI)	Rank	% (95% CI)	Rank	% (95% CI)	Rank	% (95% CI)	Rank	% (95% CI)	Rank
Hordaland	7.8 (7.6–8.0)	1	38.0 (37.0–39.1)	1	8.3 (8.1–8.6)	1	36.3 (35.0–37.8)	2	7.3 (7.1–7.6)	2	39.9 (38.4–41.3)	1
Oslo	7.3 (7.2–7.5)	2	32.9 (32.2–33.6)	4	6.9 (6.7–7.1)	2	31.8 (30.9–32.8)	5	7.8 (7.6–8.1)	1	34.3 (33.3–35.4)	4
Rogaland	5.9 (5.7–6.2)	3	31.7 (30.5–32.9)	5	5.2 (4.9–5.5)	4	29.4 (27.7–31.0)	7	6.7 (6.4–7.0)	3	34.3 (32.5–36.1)	5
Haugaland og Sunnhordland	5.4 (5.1–5.7)	4	37.5 (35.6–39.4)	2	5.5 (5.1–6.0)	3	38.2 (35.7–40.8)	1	5.3 (4.9–5.7)	4	36.6 (33.8–39.5)	2
Asker og Bærum	4.6 (4.3–4.9)	5	34.0 (32.0–36.1)	3	5.0 (4.6–5.4)	6	32.9 (30.3–35.7)	4	4.2 (3.9–4.6)	9	35.4 (32.4–38.5)	3
Søndre Buskerud	4.3 (4.1–4.6)	6	28.5 (27.1–29.9)	8	4.2 (3.9–4.5)	10	26.3 (24.5–28.2)	9	4.5 (4.2–4.8)	5	31.0 (28.9–33.1)	8
Telemark	4.3 (4.1–4.5)	7	31.3 (29.8–32.8)	6	4.4 (4.1–4.7)	9	30.7 (28.8–32.7)	6	4.3 (4.0–4.6)	8	32.1 (29.9–34.4)	6
Salten	4.3 (4.0–4.6)	8	26.4 (24.4–28.5)	10	4.2 (3.8–4.7)	11	23.8 (21.2–26.6)	13	4.4 (4.0–4.9)	6	29.4 (26.4–32.7)	9
Follo	4.2 (3.9–4.5)	9	30.8 (28.8–32.9)	7	5.0 (4.6–5.5)	5	33.1 (30.3–36.0)	3	3.5 (3.1–3.9)	12	28.1 (25.2–31.1)	10
Sør-Trøndelag	4.2 (4.0–4.4)	10	25.4 (24.3–26.5)	11	4.0 (3.8–4.3)	12	24.3 (22.9–25.8)	11	4.4 (4.1–4.6)	7	26.6 (25.0–28.3)	12
Østfold	3.9 (3.8–4.1)	11	27.2 (26.0–28.5)	9	3.8 (3.5–4.0)	13	24.0 (22.5–25.6)	12	4.1 (3.9–4.4)	10	31.4 (29.5–33.4)	7
Østfinnmark	3.9 (3.4–4.4)	12	24.9 (21.9–28.1)	13	4.8 (4.1–5.6)	7	28.1 (24.0–32.5)	8	2.9 (2.4–3.6)	14	20.2 (16.0–25.1)	17
Troms	3.7 (3.4–3.9)	13	19.2 (17.8–20.6)	17	4.5 (4.1–4.8)	8	20.9 (19.1–22.8)	15	2.9 (2.6–3.2)	15	16.9 (15.1–19.0)	21
Nordre Buskerud	3.1 (2.9–3.4)	14	25.1 (23.1–27.2)	12	3.0 (2.7–3.4)	16	23.2 (20.6–26.1)	14	3.3 (2.9–3.6)	13	27.0 (24.1–30.1)	11
Helgeland	3.0 (2.8–3.3)	15	23.0 (20.9–25.3)	14	3.5 (3.1–3.9)	14	25.1 (22.2–28.2)	10	2.5 (2.2–2.9)	18	20.3 (17.3–23.6)	16
Vestfold	2.9 (2.7–3.0)	16	20.5 (19.4–21.7)	16	2.3 (2.1–2.5)	20	16.0 (14.6–17.5)	20	3.5 (3.3–3.8)	11	26.4 (24.5–28.4)	13
Sogn og Fjordane	2.7 (2.5–2.9)	17	22.2 (20.3–24.3)	15	2.6 (2.4–2.9)	17	20.8 (18.3–23.4)	16	2.8 (2.5–3.1)	16	24.0 (21.2–27.2)	14
Vestfinnmark	2.7 (2.3–3.1)	18	16.4 (14.2–18.8)	20	3.2 (2.7–3.9)	15	18.7 (15.7–22.1)	18	2.1 (1.7–2.6)	21	13.3 (10.4–16.8)	24
Romerike	2.3 (2.2–2.5)	19	14.4 (13.4–15.5)	22	1.9 (1.7–2.1)	22	11.4 (10.2–12.8)	24	2.7 (2.5–2.9)	17	17.5 (16.0–19.1)	20
Midtre Hålogaland	2.1 (2.0–2.3)	20	18.6 (17.0–20.3)	18	2.4 (2.2–2.7)	18	19.2 (17.2–21.5)	17	1.8 (1.6–2.1)	22	17.7 (15.3–20.3)	19
Nord-Trøndelag	2.1 (1.9–2.2)	21	17.6 (16.0–19.2)	19	1.9 (1.7–2.1)	23	16.7 (14.7–18.8)	19	2.2 (2.0–2.5)	20	18.6 (16.4–21.1)	18
Nordmøre og Romsdal	1.8 (1.7–2.0)	22	16.3 (14.7–18.0)	21	1.4 (1.2–1.6)	24	11.6 (9.8–3.7)	23	2.3 (2.0–2.6)	19	22.0 (19.3–24.9)	15
Agder	1.7 (1.6–1.8)	23	12.6 (11.7–13.4)	25	2.3 (2.2–2.5)	19	15.4 (14.2–16.7)	21	1.1 (1.0–1.2)	27	9.0 (8.0–10.2)	27
Vestoppland	1.6 (1.4–1.7)	24	12.6 (11.3–14.0)	24	1.4 (1.2–1.6)	25	10.5 (8.9–12.2)	25	1.8 (1.6–2.1)	23	15.1 (13.1–17.4)	22
Hedmark	1.6 (1.5–1.7)	25	12.7 (11.7–13.8)	23	2.0 (1.8–2.2)	21	15.0 (13.6–16.5)	22	1.1 (1.0–1.3)	26	9.8 (8.5–11.2)	26
Sunnmøre	1.0 (0.9–1.2)	26	8.8 (7.7–10.1)	26	0.6 (0.4–0.7)	27	4.2 (3.2–5.5)	27	1.5 (1.3–1.7)	24	13.9 (12.0–16.1)	23
Gudbrandsdal	0.9 (0.8–1.0)	27	7.9 (6.7–9.4)	27	0.6 (0.5–0.8)	26	4.6 (3.4–6.2)	26	1.2 (1.0–1.5)	25	12.2 (9.9–14.9)	25

Proportion of deaths undergoing forensic autopsy in Norway 1996–2017. Data from the Norwegian Cause of Death Registry, 1996–2017, for the periods 1996–2017, 1996–2006 and 2007-2017, in all ages combined and age at death <60 years of age.

CI: confidence interval.

### Municipalities and distance to autopsy facilities

Municipalities with more than 50,000 residents had a higher autopsy proportion (5.7%) compared to smaller municipalities (3.0%). The same holds for the most centrally located municipalities (5.3% compared to 2.8% in the rest) and those situated <50 km from the autopsy facilities (5.7% compared to 2.9% in the rest). Apart from this, we did not find a clear gradient within the smaller or more rural municipalities.

### Group-wise analyses

A summary of the findings is presented in Table III. We performed separate analyses for the eight cause-of-death groups. For deaths due to natural causes, accidental poisonings and other external causes, the (type of) place of death was the most important factor influencing autopsy, with a low proportion in deaths in health-care institutions. For ill-defined causes of death and accidental falls, age was the most important factor, with the proportion of autopsies falling steeply at ages >60. For deaths due to traffic accidents and suicides, the police district was the most important explanatory factor. For homicides, almost all deaths underwent autopsy, and none of the explanatory factors were associated with the use of forensic autopsy. The exception was (type of) place of death, with fewer autopsies of deaths in nursing homes. However, the numbers are very small (two out of four deaths classified as homicides). It is noteworthy that the police district was among the top three explanatory factors in all cause-of-death groups (homicides excluded), whereas variables related to population size, the rurality of the municipality and distance to the autopsy facility seemed to have only a minor influence. For detailed results, see the Supplemental Material.

**Table III.** Summary of group-wise logistic regression.

Autopsy frequency (%)	Natural causes of death		Ill-defined causes of death		Traffic accidents		Accidental falls		Accidental poisonings		Other external causes of death		Suicides		Homicides	
	1.7		3.0		52.3		5.2		78.9		39.6		63.7		96.6	
	LR stat.	*p*-Value	LR stat.	*p*-Value	LR stat.	*p*-Value	LR stat.	*p*-Value	LR stat.	*p*-Value	LR stat.	*p*-Value	LR stat.	*p*-Value	LR stat.	*p*-Value
Year of death	264	<0.001	72	<0.001	31	<0.001	5	0.09	8	0.02	4	0.11	74	<0.001	3	0.21
Sex	220	<0.001	5	0.03	2	0.18	9	<0.001	3	0.10	0.3	0.60	37	<0.001	0.4	0.55
Age group	15033	<0.001	1426	<0.001	9	0.40	1082	<0.001	385	<0.001	706	<0.001	160	<0.001	10	0.33
Place of death	27603	<0.001	266	<0.001	300	<0.001	886	<0.001	1010	<0.001	1728	<0.001	585	<0.001	13	0.01
Police district	2820	<0.001	356	<0.001	843	<0.001	233	<0.001	454	<0.001	353	<0.001	1847	<0.001	33	0.16
Population	503	<0.001	61	<0.001	12	0.04	11	0.06	6	0.47	9	0.10	26	<0.001	5	0.45
Centrality	61	<0.001	5	0.49	8	0.27	6	0.43	10	0.14	5	0.61	9	0.19	7	0.28
Distance	17	0.08	28	0.002	29	<0.001	11	0.35	9	0.51	17	0.07	57	<0.001	11	0.37

Likelihood ratio statistic (rounded) and *p*-value for each explanatory variable. The likelihood ratio statistic (–2 logL) is computed by comparing the full model to the model *without* the variable in question. The higher the LR statistics, the more the model is weakened by excluding the variable in question.

For detailed description, see supplementary material.

Data from The Norwegian Cause of Death Registry, 1996–2017.

Age: 10-year groups; place of death: five categories; police districts: *N*=27; population size: six groups; centrality index: seven levels; distance from place of death to autopsy facility: 50 km groups.

## Discussion

In this population-based retrospective observational study, we used data from the NCoDR for the years 1996–2017 to investigate factors that might influence the utilisation of forensic autopsies. In the analyses, we used logistic regression, divided into groups by the registered cause of death. The proportion of forensic autopsies varied greatly with the cause of death. Overall, the three most important explanatory factors across the strata were the (type of) place of death, followed by the police district where the death took place and the age of the deceased.

### Strength and limitations

The major strength of the study is the population-based design using individual data for >98% of Norwegian residents dying in Norway in the study period. The coverage and quality of demographic data in the NCoDR is very good, and the quality of medical data, such as the underlying cause of death, is also considered good [14]. Although the reporting of autopsy results to the NCoDR is compulsory, there is some discrepancy between data from the NCoDR, the Norwegian Board of Forensic Medicine and the Norwegian Society of Pathology. Some of this discrepancy is due to deaths of non-residents not included in the NCoDR. Also, failure to report from the autopsy departments and erroneous registration of medical versus forensic autopsies at the NCoDR may contribute. We estimate that around 5% of the forensic autopsy reports are missing in our data, contributing to a slight underestimation of the proportion of forensic autopsies. If the data are not missing at random, this could introduce bias in the results.

The perceived cause of death is the major determinant for whether the physician viewing the body decides to notify the police, and this is equally important when the police decide to request a forensic autopsy. To date, neither the NCoDR nor the Norwegian Police Directorate has comprehensive figures for how many deaths are reported from physicians to the police. If a notifiable death is not sent for autopsy, in principle, we cannot tell whether this is because the police have not been notified by the doctor or if the police have declined the autopsy. The very large variation between police districts suggests that factors relating to local procedures and attitudes of the police are important.

We do not know the physician’s initial assessment, and the registered cause of death in the NCoDR is influenced by the autopsy results (or lack thereof). Using the registered cause of death as an explanatory variable in the logistic regression might thus be methodologically unsound. To estimate the impact of the other explanatory variables in different scenarios, we divided the data according to the underlying cause of death. A major limitation of this study is that the registered cause of death might be wrong, especially when no autopsy has been performed. Indeed, classification of cause of death to the ill-defined group might be the result of a lack of autopsy, as shown by Ylijoki-Sørensen et al. [24]. Our study was not designed to ascertain misclassification due to a lack of autopsy.

For some characteristics, we noticed separation, with all observations falling into the same group (autopsy proportion either 0% or 100%). This can introduce problems in the estimation of the coefficients, giving very large confidence intervals. To avoid this, we used Firth’s bias reduction in the regressions [23].

### Discussion of results

The explanatory variables can be divided into three main groups.

#### Factors related to the cause and circumstances around the death

One could argue that the only legitimate factors when requesting a forensic autopsy are the circumstances and perceived cause of death. We would expect a variation in the autopsy proportion between different causes of death as well as the (type of) place of death. Essentially all homicides, but only 1.7% of deaths from natural causes are sent for autopsy. Hasselqvist and Rammer found that 7.5% of the homicides in Sweden were not discovered until autopsy [25]. Even in deaths from external causes, few cases undergo autopsy if the death occurs in a health-care institution, probably reflecting more information about the injuries and circumstances.

#### Demographic factors – age and sex

The proportion of autopsies falls steeply with age. This can in part be explained by a higher frequency of external causes of death in the young. However, in several cause-of-death groups, age is an important explanatory factor, even in the multi-predictor models. In accidental falls, the largest group is low-level, low-energy falls in the elderly [26]. We believe that many of these deaths are not reported to the police, and even if the police are notified, an autopsy is seldom requested. The age gradient in accidental poisonings and suicides might be more problematic, as investigating deaths in the elderly should be as important as in the young. More than twice as many men as women underwent forensic autopsy, but men are more likely than women to suffer an external cause of death. In the group-wise, multi-predictor regressions, sex was among the least important factors.

#### Geographic factors – police district and municipalities

In all groups, police district was among the top three explanatory factors. Within some cause-of-death groups, notably traffic accidents and suicides, the variation in autopsy proportion between districts was very large (Figure 2). In traffic accidents, the range was from 6.5% to 87.2%. This observation may reflect a number of more or less unidentified factors, including local attitudes, habits, procedures, economic priorities and so on. One aspect could be attitudes towards the purpose of investigating deaths. Is the forensic procedure viewed as a means to examine possible criminal cases only, or does the task include public health, preventive measures, the relatives’ needs, and cause-of-death statistics? We also speculate that a close communication between the police authorities and, on the one hand, the doctors in the community reporting deaths and, on the other hand, the forensic pathologists performing the autopsies could stimulate a broader understanding of the different goals of an autopsy. In 2016, the number of police districts was reduced from 27 to 12, and in 2020, compulsory forensic autopsy of all traffic deaths was introduced. Time will tell if these changes will reduce the geographic variation in forensic autopsies.

Currently, >95% of forensic autopsies in Norway are performed in Oslo, Bergen, Trondheim, Tromsø and Stavanger. The expenditure for a forensic autopsy consists partly of the transport to the autopsy facilities, and this must be covered by the requesting police district. When the distance is substantial, the transport costs may supersede the fee for the autopsy itself. In the unstratified introductory analyses, there was a tendency for the autopsy proportion to be higher in the large and most central municipalities, closest to the autopsy facilities, but in the group-wise, multi-predictor models, these factors had a low influence, contrary to common belief. In some strata, the effect was not statistically significant; in others, the influence was minor compared to other factors. Some police districts with large transport distances have higher autopsy frequencies than districts close to the autopsy facility (Figure 1).

### Implications of the study

The two major areas of implications concern the protection of the legal rights of the individual and trust in the judicial system, and the quality of the cause-of-death statistics. Ideally, the decision about starting an investigation should be influenced solely by the circumstances around the death (or the discovery of the body). If unjustified differences in the frequency of autopsies lead to insufficient investigation of possible unnatural deaths, this may in worst-case scenarios mean that criminal cases remain undetected. As the results from forensic autopsies are important sources for cause-of-death statistics, variations in autopsy frequency might lead to suboptimal quality of statistics and introduce spurious shifts (e.g. over time or between geographical regions). As a result, this could lead to misleading information for surveillance, quality analysis, prevention and research.

## Supplemental Material

sj-pdf-1-sjp-10.1177_1403494821997208 – Supplemental material for Forensic autopsies in Norway 1996–2017: A retrospective study of factors associated with deaths undergoing forensic autopsy Click here for additional data file. Supplemental material, sj-pdf-1-sjp-10.1177_1403494821997208 for Forensic autopsies in Norway 1996–2017: A retrospective study of factors associated with deaths undergoing forensic autopsy by Christian Lycke Ellingsen, G. Cecilie Alfsen and Geir Sverre Braut in Scandinavian Journal of Public Health
