# Supplementary material for: Forensic autopsies in Norway 1996–2017: A retrospective study of factors associated with deaths undergoing forensic autopsy
Source: Scand J Public Health. 2021 Mar 8;50(4):424–31. doi: 10.1177/1403494821997208 (PMC9152604; doi:10.1177/1403494821997208)
Supplement: sj-pdf-1-sjp-10.1177_1403494821997208 – Supplemental material for Forensic autopsies in Norway 1996–2017: A retrospective study of factors associated with deaths undergoing forensic autopsy [file sj-pdf-1-sjp-10.1177_1403494821997208.pdf]

Forensic autopsies in Norway 1996-2017 – a retrospective study of factors correlated with the autopsy frequency

Supplementary tables

Notifiable deaths in Norway

**Deaths in Norway where the police is to be notified**<br>(regulation FOR-2000-12-21-1378)
Homicide or other forms for assault*
Suicide or self-inflicted injury
Accidents (e.g. fire, avalanche, lightning, drowning, fall, transport accident)
Occupational accident or injury
Medical misadventure, accident or neglect
Drug abuse
Sudden and unexpected death of unknown cause
Deaths in custody
Unidentified body*
Children below 18 years with unknown cause of death, dying outside health care institution*<br>(regulation FOR-1985-06-28-1679, §13-2)
(*Autopsy mandatory)

Definitions

**Underlying cause of death**	**ICD-10 codes**
1. Natural	A00-Q99 (except F11-F12, F14-F16, F19), R95
2. Ill-defined	R00-R99 (except R95)
3. Traffic accidents	V00-V89, Y85.0
4. Accidental falls	W00-W19, X59 in combination with S72
5. Accidental poisonings	X40-X49, F11-F12, F14-F16, F19
6. Other accidents and events of undetermined intent	V90 – V99, W20 – X39.9, X50 – X59, Y10 – Y84, Y85.9 – Y86, Y87.2 – Y89.9
7. Intentional self-harm (suicide)	X60 – X84, Y87.0
8. Assault (homicide)	X85 – Y09, Y87.1
**Place of death**	
1. At home	At home
2. Hospital	Somatic and psychiatric hospitals
3. Nursing home	Nursing homes, other health care institutions
4. Other known	Other known, during transport
5. Unknown	Unknown, abroad

Overview of results

All causes of death

	All deaths	Forensic autopsies	Per cent
**Total**	**920232**	**37398**	**4.1**

Variable	Level	All deaths	Forensic autopsies	Per cent	Autopsies per 100,000
**Year**	1996	43636	1813	4.2	41.4
	1997	44373	1940	4.4	44.0
	1998	43966	1972	4.5	44.5
	1999	44834	1775	4.0	39.8
	2000	43682	1884	4.3	42.0
	2001	43666	1715	3.9	38.0
	2002	44036	1734	3.9	38.2
	2003	42177	1626	3.9	35.6
	2004	40816	1600	3.9	34.9
	2005	40759	1599	3.9	34.6
	2006	40872	1552	3.8	33.3
	2007	41556	1584	3.8	33.6
	2008	41300	1607	3.9	33.7
	2009	40950	1621	4.0	33.6
	2010	40934	1595	3.9	32.6
	2011	40759	1659	4.1	33.5
	2012	41349	1531	3.7	30.5
	2013	40475	1630	4.0	32.1
	2014	39728	1652	4.2	32.2
	2015	40075	1812	4.5	34.9
	2016	40128	1733	4.3	33.1
	2017	40161	1764	4.4	33.4
**Sex**	Male	446396	26648	6.0	
	Female	473836	10750	2.3	
**Age group**	0-9	5634	1045	18.6	
	10-19	3009	1429	47.5	
	20-29	7536	4484	59.5	
	30-39	10865	5170	47.6	
	40-49	22138	6196	28.0	
	50-59	51013	7082	13.9	
	60-69	100033	5918	5.9	
	70-79	202944	3791	1.9	
	80-89	344250	1997	0.6	
	90+	172810	286	0.2	
**Place of death**	At_home	139839	18006	12.9	
	Hospital	343972	4530	1.3	
	Nursing_home	393353	437	0.1	
	Other_known	29003	10508	36.2	

Level	All deaths	Forensic autopsies	Per cent
1996-2002	308193	12833	4.2
2003-2010	329364	12784	3.9
2011-2017	282675	11781	4.2

	Unknown	14065	3917	27.9	
**Cause of death**	1.Natural	830154	14339	1.7	
	2.Ill-defined	30074	889	3.0	
	3.Traffic_acc	5631	2946	52.3	
	4.Acc_falls	20306	1050	5.2	
	5.Acc_poisonings	7719	6090	78.9	
	6.Other_ext	9094	3601	39.6	
	7.Suicide	11984	7639	63.7	
	8.Homicide	874	844	96.6	
	Missing	4396	0	0.0	
**Police district**	Agder	52658	906	1.7	
	Asker og Bærum	22681	1041	4.6	
	Follo	17782	751	4.2	
	Gudbrandsdal	17935	160	0.9	
	Haugaland og Sunnhordland	24705	1334	5.4	
	Hedmark	48116	754	1.6	
	Helgeland	15874	481	3.0	
	Hordaland	77839	6096	7.8	
	Midtre Hålogaland	26694	570	2.1	
	Nord-Trøndelag	27846	572	2.1	
	Nordmøre og Romsdal	23753	433	1.8	
	Nordre Buskerud	18894	593	3.1	
	Oslo	108425	7955	7.3	
	Rogaland	45987	2736	5.9	
	Romerike	41148	959	2.3	
	Salten	15861	684	4.3	
	Sogn og Fjordane	22019	595	2.7	
	Sunnmøre	24738	253	1.0	
	Søndre Buskerud	37658	1637	4.3	
	Sør-Trøndelag	54320	2277	4.2	
	Telemark	38780	1673	4.3	
	Troms	22870	842	3.7	
	Vestfinnmark	7323	195	2.7	
	Vestfold	43670	1257	2.9	
	Vestoppland	25988	412	1.6	
	Østfinnmark	6234	242	3.9	
	Østfold	50434	1990	3.9	
**Municipality population**	<2000	24640	860	3.5	
	2000-4999	81893	2943	3.6	
	5000-9999	107207	3288	3.1	
	10000-19999	137192	4308	3.1	
	20000-49999	221488	6049	2.7	(0-49999 collated 3.0 %)
	50000+	347812	19950	5.7	
**Urbanity index**	0B (Most rural)	95353	2903	3	
	0A	23694	865	3.7	
	1B	38087	1030	2.7	

	1A	36127	1057	2.9	
	2B	72187	1739	2.4	
	2A	192061	5400	2.8	(0B-2A collated 2.8%)
	3A (Most urban)	462723	24404	5.3	
**Distance to autopsy facility (km)**	0-49	383673	21944	5.7	
	50-99	143128	5053	3.5	
	100-149	159915	4486	2.8	
	150-199	62029	2182	3.5	
	200-249	35361	973	2.8	
	250-299	26809	472	1.8	
	300-349	45282	897	2.0	
	350-399	27045	472	1.8	
	400-449	16387	332	2.0	
	450-499	13328	335	2.5	
	> 500	7275	252	3.5	(50-500+ collated 2.9%)

Stratified logistic regression

1. Natural causes of death

		Deaths	Forensic autopsies	Per cent
**Total**		**830154**	**14339**	**1.7**

Variable	Level	Deaths	For.aut.	Per cent	OR (univariate)	95% CI	LRT (univariate)	p value	OR (multi predictor)	95% CI	LRT (multi predictor)	p value
**Year**	1996-2002	279172	4907	1.8	1	Ref.	48.4	< 0.001	1	Ref.	264.4	< 0.001
	2003-2010	297300	4763	1.6	0.91	(0.87-0.95)			1.02	(0.97-1.07)		
	2011-2017	253682	4669	1.8	1.05	(1.01-1.09)			1.42	(1.36-1.49)		
**Sex**	Male	398889	10060	2.5	1	Ref.	2920	< 0.001	1	Ref.	220.2	< 0.001
	Female	431265	4279	1.0	0.39	(0.37-0.40)			0.73	(0.70-0.77)		
**Age**	20-29	1887	391	20.7	1	Ref.	28075	< 0.001	1	Ref.	15033	< 0.001
	0-9	5117	761	14.9	0.67	(0.58-0.77)			1.08	(0.92-1.28)		
	10-19	1045	183	17.5	0.81	(0.67-0.99)			0.87	(0.68-1.10)		
	30-39	4890	841	17.2	0.79	(0.70-0.91)			0.79	(0.67-0.94)		
	40-49	15671	1953	12.5	0.54	(0.48-0.61)			0.47	(0.40-0.54)		
	50-59	44050	3333	7.6	0.31	(0.28-0.35)			0.26	(0.23-0.30)		
	60-69	93084	3562	3.8	0.15	(0.14-0.17)			0.14	(0.12-0.16)		
	70-79	191927	2225	1.2	0.04	(0.04-0.05)			0.05	(0.05-0.06)		
	80-89	319518	960	0.3	0.01	(0.01-0.01)			0.02	(0.02-0.02)		
	90 and above	152965	130	0.1	0.003	(0-0.004)			0.01	(0.01-0.01)		
**Place of death**	At_home	114106	8848	7.8	1	Ref.	36752	< 0.001	1	Ref.	27602	< 0.001
	Hospital	325663	1829	0.6	0.07	(0.06-0.07)			0.04	(0.04-0.04)		
	Nursing_home	366813	214	0.1	0.01	(0.01-0.01)			0.02	(0.01-0.02)		
	Other_known	13787	2096	15.2	2.13	(2.03-2.24)			1.92	(1.80-2.03)		
	Unknown	9785	1352	13.8	1.91	(1.79-2.03)			2.10	(1.95-2.26)		
**Police district**	Agder	47483	255	0.5	1	Ref.	6036	< 0.001	1	Ref.	2820	< 0.001
	Asker og Bærum	20569	388	1.9	3.56	(3.04-4.17)			3.08	(2.32-4.08)		
	Follo	15759	218	1.4	2.60	(2.17-3.12)			2.73	(2.03-3.68)		
	Gudbrandsdal	16145	56	0.3	0.65	(0.48-0.86)			1.14	(0.77-1.67)		
	Haugaland og Sunnhordland	22500	505	2.2	4.25	(3.66-4.95)			8.09	(6.26-10.48)		
	Hedmark	43188	251	0.6	1.08	(0.91-1.29)			1.93	(1.47-2.53)		
	Helgeland	14326	162	1.1	2.12	(1.74-2.58)			3.59	(2.59-4.97)		
	Hordaland	69835	3009	4.3	8.32	(7.34-9.49)			12.57	(9.7-16.33)		
	Midtre Hålogaland	24258	197	0.8	1.52	(1.26-1.83)			2.69	(2.11-3.43)		
	Nord-Trøndelag	25223	225	0.9	1.67	(1.39-2.00)			2.36	(1.79-3.09)		
	Nordmøre og Romsdal	21457	133	0.6	1.16	(0.94-1.42)			1.87	(1.39-2.51)		
	Nordre Buskerud	16685	170	1.0	1.91	(1.57-2.32)			3.00	(2.23-4.04)		
	Oslo	95933	3048	3.2	6.07	(5.35-6.91)			5.95	(4.59-7.74)		
	Rogaland	41720	1069	2.6	4.86	(4.25-5.59)			5.46	(4.20-7.13)		
	Romerike	37429	321	0.9	1.60	(1.36-1.89)			2.33	(1.75-3.10)		
	Salten	14237	240	1.7	3.18	(2.66-3.79)			4.90	(3.50-6.86)		
	Sogn og Fjordane	20349	215	1.1	1.98	(1.65-2.37)			3.35	(2.57-4.37)		
	Sunnmøre	22806	78	0.3	0.64	(0.49-0.82)			1.03	(0.74-1.43)		

	Søndre Buskerud	34027	611	1.8	3.38	(2.93-3.92)			4.03	(3.09-5.28)		
	Sør-Trøndelag	48804	860	1.8	3.32	(2.89-3.82)			3.71	(2.85-4.85)		
	Telemark	35241	600	1.7	3.20	(2.77-3.72)			5.11	(3.98-6.58)		
	Troms	20680	294	1.4	2.67	(2.26-3.16)			2.49	(1.88-3.30)		
	Vestfinnmark	6529	49	0.8	1.41	(1.03-1.90)			1.47	(1.01-2.13)		
	Vestfold	39719	418	1.1	1.97	(1.69-2.30)			3.44	(2.62-4.52)		
	Vestoppland	23346	123	0.5	0.98	(0.79-1.22)			1.58	(1.17-2.14)		
	Østfinnmark	5607	80	1.4	2.69	(2.08-3.45)			6.50	(3.65-11.67)		
	Østfold	46299	764	1.7	3.10	(2.70-3.58)			4.15	(3.16-5.46)		
**Municipality population**	< 2000	21851	284	1.3	1	Ref.	2414	< 0.001	1	Ref.	503.5	< 0.001
	2000-4999	72689	1041	1.4	1.10	(0.97-1.26)			0.94	(0.81-1.09)		
	5000-9999	95302	1123	1.2	0.90	(0.79-1.03)			1.01	(0.87-1.19)		
	10000-19999	124273	1455	1.2	0.90	(0.79-1.02)			1.01	(0.86-1.19)		
	20000-49999	202375	2171	1.1	0.82	(0.73-0.93)			1.36	(1.16-1.61)		
	> 50000	313664	8265	2.6	2.05	(1.83-2.32)			2.36	(2.00-2.79)		
**Urbanity index**	0B (Most rural)	85268	997	1.2	1	Ref.	1974	< 0.001	1	Ref.	60.5	< 0.001
	0A	20922	315	1.5	1.29	(1.14-1.47)			1.01	(0.85-1.19)		
	1B	34799	388	1.1	0.95	(0.85-1.07)			1.58	(1.35-1.84)		
	1A	32816	360	1.1	0.94	(0.83-1.06)			1.27	(1.06-1.52)		
	2B	65772	596	0.9	0.77	(0.70-0.86)			1.22	(1.05-1.42)		
	2A	175243	1938	1.1	0.94	(0.88-1.02)			0.99	(0.85-1.16)		
	3A (Most urban)	415334	9745	2.3	2.03	(1.90-2.17)			1.35	(1.14-1.59)		
**Distance to autopsy facility (km)**	0-49	344784	8943	2.6	1	Ref.	2932	< 0.001	1	Ref.	16.7	0.08
	50-99	129393	1800	1.4	0.53	(0.50-0.56)			0.90	(0.82-1.00)		
	100-149	144691	1601	1.1	0.42	(0.40-0.44)			0.96	(0.84-1.11)		
	150-199	55959	768	1.4	0.52	(0.49-0.56)			1.08	(0.91-1.29)		
	200-249	31962	356	1.1	0.42	(0.38-0.47)			1.11	(0.90-1.37)		
	250-299	24281	137	0.6	0.21	(0.18-0.25)			1.00	(0.76-1.32)		
	300-349	41197	299	0.7	0.27	(0.24-0.31)			0.97	(0.75-1.27)		
	350-399	24521	143	0.6	0.22	(0.19-0.26)			0.91	(0.65-1.27)		
	400-449	14709	102	0.7	0.26	(0.22-0.32)			0.88	(0.62-1.24)		
	450-499	12091	108	0.9	0.34	(0.28-0.41)			0.83	(0.57-1.21)		
	> 500	6566	82	1.2	0.48	(0.38-0.59)			0.55	(0.30-0.97)		

2. Ill-defined causes of death

	Deaths	Forensic autopsies	Per cent
**Total**	**30074**	**889**	**3.0**

Variable	Level	Deaths	For.aut.	Per cent	OR (univariate)	95% CI	LRT (univariate)	p value	OR (multi predictor)	95% CI	LRT (multi predictor)	p value
**Year**	1996-2002	10049	397	4.0	1	Ref.	50.9	< 0.001	1	Ref.	71.5	< 0.001
	2003-2010	10642	250	2.3	0.59	(0.50-0.69)			0.47	(0.39-0.57)		
	2011-2017	9383	242	2.6	0.64	(0.55-0.76)			0.54	(0.45-0.66)		
**Sex**	Male	11842	538	4.5	1	Ref.	166.5	< 0.001	1	Ref.	4.67	0.03
	Female	18232	351	1.9	0.41	(0.36-0.47)			1.21	(1.02-1.43)		
**Age**	20-29	81	55	67.9	1	Ref.	1926	< 0.001	1	Ref.	1425	< 0.001
	0-9	35	20	57.1	0.63	(0.28-1.42)			0.39	(0.15-1.03)		
	10-19	26	19	73.1	1.24	(0.49-3.41)			2.41	(0.78-8.18)		
	30-39	165	72	43.6	0.37	(0.21-0.64)			0.38	(0.19-0.74)		
	40-49	410	108	26.3	0.17	(0.10-0.28)			0.09	(0.05-0.17)		
	50-59	1144	154	13.5	0.07	(0.04-0.12)			0.04	(0.02-0.07)		
	60-69	1981	96	4.8	0.02	(0.01-0.04)			0.008	(0-0.02)		
	70-79	4312	173	4.0	0.02	(0.01-0.03)			0.004	(0-0.008)		
	80-89	10764	163	1.5	0.01	(0 -0.01)			0.003	(0-0.005)		
	90 and above	11156	29	0.3	0.001	(0-0.002)			0.001	(0-0.003)		
**Place of death**	At_home	11275	658	5.8	1	Ref.	1207	< 0.001	1	Ref.	266.2	< 0.001
	Hospital	925	23	2.5	0.42	(0.27-0.62)			0.30	(0.17-0.51)		
	Nursing_home	14948	8	0.1	0.01	(0-0.02)			0.04	(0.02-0.08)		
	Other_known	1817	92	5.1	0.86	(0.69-1.08)			0.61	(0.46-0.80)		
	Unknown	1109	108	9.7	1.75	(1.40-2.15)			1.75	(1.33-2.29)		
**Police district**	Agder	1836	14	0.8	1	Ref.	750.4	< 0.001	1	Ref.	356.4	< 0.001
	Asker og Bærum	646	10	1.5	2.07	(0.91-4.58)			1.49	(0.33-7.19)		
	Follo	690	8	1.2	1.57	(0.64-3.60)			2.24	(0.47-11.1)		
	Gudbrandsdal	644	5	0.8	1.08	(0.37-2.75)			1.06	(0.17-6.64)		
	Haugaland og Sunnhordland	620	5	0.8	1.12	(0.38-2.86)			4.30	(0.90-20.6)		
	Hedmark	1681	14	0.8	1.09	(0.52-2.29)			2.47	(0.63-10.5)		
	Helgeland	452	2	0.4	0.70	(0.14-2.28)			0.65	(0.07-4.97)		
	Hordaland	2929	307	10.5	14.74	(9.01-26.21)			16.8	(4.37-71.3)		
	Midtre Hålogaland	784	8	1.0	1.38	(0.56-3.16)			3.70	(0.94-13.8)		
	Nord-Trøndelag	998	19	1.9	2.50	(1.27-5.05)			10.8	(2.81-44.9)		
	Nordmøre og Romsdal	925	3	0.3	0.48	(0.12-1.38)			1.24	(0.21-6.64)		
	Nordre Buskerud	850	6	0.7	0.97	(0.36-2.36)			2.35	(0.46-12.2)		
	Oslo	3977	221	5.6	7.41	(4.51-13.23)			4.28	(1.11-18.2)		
	Rogaland	1262	56	4.4	5.89	(3.38-10.93)			6.27	(1.59-27.2)		
	Romerike	1106	21	1.9	2.49	(1.28-4.97)			2.90	(0.68-13.3)		
	Salten	472	19	4.0	5.40	(2.73-10.94)			15.2	(2.32-105.9)		
	Sogn og Fjordane	516	5	1.0	1.35	(0.46-3.44)			3.06	(0.55-16.0)		
	Sunnmøre	619	0	0	0.10	(0-0.76)			0.06	(0-0.80)		
	Søndre Buskerud	1223	27	2.2	2.89	(1.55-5.63)			4.99	(1.22-22.1)		
	Sør-Trøndelag	2167	35	1.6	2.09	(1.16-3.99)			1.59	(0.39-7.04)		

	Telemark	1217	23	1.9	2.47	(1.29-4.89)			4.57	(1.24-18.5)		
	Troms	573	15	2.6	3.49	(1.69-7.26)			2.15	(0.50-9.89)		
	Vestfinnmark	221	3	1.4	2.01	(0.52-5.88)			7.71	(1.27-40.8)		
	Vestfold	1191	21	1.8	2.31	(1.19-4.61)			7.41	(1.86-32.4)		
	Vestoppland	1040	6	0.6	0.79	(0.29-1.92)			2.02	(0.42-9.84)		
	Østfinnmark	167	3	1.8	2.67	(0.69-7.83)			65.4	(6.03-832.7)		
	Østfold	1268	33	2.6	3.41	(1.87-6.53)			6.97	(1.65-31.9)		
**Municipality population**	< 2000	1064	10	0.9	1	Ref.	516.5	< 0.001	1	Ref.	60.8	< 0.001
	2000-4999	3640	30	0.8	0.85	(0.43-1.8)			0.92	(0.40-2.27)		
	5000-9999	4412	43	1.0	1.00	(0.53-2.08)			1.21	(0.52-3.01)		
	10000-19999	4361	69	1.6	1.63	(0.89-3.31)			1.25	(0.54-3.13)		
	20000-49999	5597	88	1.6	1.61	(0.89-3.26)			1.20	(0.50-3.10)		
	> 50000	11000	649	5.9	6.30	(3.6-12.43)			4.38	(1.85-11.2)		
**Urbanity index**	0B (Most rural)	3799	33	0.9	1	Ref.	310.6	< 0.001	1	Ref.	5.41	0.49
	0A	1242	12	1.0	1.14	(0.57-2.14)			1.64	(0.61-4.26)		
	1B	982	11	1.1	1.33	(0.65-2.53)			0.72	(0.27-1.83)		
	1A	1137	18	1.6	1.86	(1.03-3.25)			1.72	(0.63-4.66)		
	2B	2039	27	1.3	1.54	(0.92-2.55)			1.43	(0.59-3.46)		
	2A	5335	81	1.5	1.74	(1.18-2.65)			0.97	(0.39-2.46)		
	3A (Most urban)	15540	707	4.5	5.36	(3.85-7.74)			1.21	(0.46-3.22)		
**Distance to autopsy facility (km)**	0-49	12431	686	5.5	1	Ref.	500.5	< 0.001	1	Ref.	27.7	0.002
	50-99	4562	72	1.6	0.28	(0.21-0.35)			0.61	(0.35-1.05)		
	100-149	5303	62	1.2	0.20	(0.16-0.26)			0.61	(0.29-1.29)		
	150-199	2264	25	1.1	0.19	(0.13-0.28)			1.08	(0.40-2.90)		
	200-249	1272	11	0.9	0.16	(0.08-0.27)			1.35	(0.39-4.50)		
	250-299	939	6	0.6	0.12	(0.05-0.24)			0.47	(0.09-2.20)		
	300-349	1324	13	1.0	0.18	(0.10-0.29)			0.53	(0.13-2.31)		
	350-399	931	3	0.3	0.06	(0.02-0.16)			1.56	(0.22-10.1)		
	400-449	517	3	0.6	0.12	(0.03-0.29)			0.87	(0.12-6.33)		
	450-499	316	7	2.2	0.41	(0.18-0.80)			2.58	(0.33-21.0)		
	> 500	215	1	0.5	0.12	(0.01-0.43)			0.04	(0-0.66)		

3. Traffic accidents

		Deaths	Forensic autopsies	Per cent
**Total**		**5631**	**2946**	**52.3**

Variable	Level	Deaths	For.aut.	Per cent	OR (univariate)	95% CI	LRT (univariate)	p value	OR (multi predictor)	95% CI	LRT (multi predictor)	p value
**Year**	1996-2002	2398	1228	51.2	1	Ref.	30.2	< 0.001	1	Ref.	30.9	< 0.001
	2003-2010	2094	1041	49.7	0.94	(0.84-1.06)			0.87	(0.76-1.00)		
	2011-2017	1139	677	59.4	1.40	(1.21-1.61)			1.42	(1.20-1.69)		
**Sex**	Male	4134	2159	52.2	1	Ref.	0.05	0.82	1	Ref.	1.76	0.18
	Female	1497	787	52.6	1.01	(0.90-1.14)			1.10	(0.96-1.26)		
**Age**	20-29	1112	615	55.3	1	Ref.	29.1	< 0.001	1	Ref.	9.2	0.41
	0-9	156	73	46.8	0.71	(0.51-0.99)			0.70	(0.47-1.04)		
	10-19	729	404	55.4	1.00	(0.83-1.21)			1.01	(0.81-1.25)		
	30-39	686	377	55.0	0.99	(0.81-1.19)			1.10	(0.88-1.37)		
	40-49	643	352	54.7	0.98	(0.80-1.19)			1.04	(0.83-1.30)		
	50-59	610	313	51.3	0.85	(0.70-1.04)			0.92	(0.73-1.16)		
	60-69	543	269	49.5	0.79	(0.65-0.97)			0.93	(0.73-1.17)		
	70-79	641	297	46.3	0.70	(0.57-0.85)			0.89	(0.70-1.11)		
	80-89	461	227	49.2	0.78	(0.63-0.97)			1.07	(0.82-1.39)		
	90 and above	50	19	38.0	0.50	(0.28-0.88)			0.67	(0.34-1.30)		
**Place of death**	At_home	85	31	36.5	1	Ref.	158.5	< 0.001	1	Ref.	295.9	< 0.001
	Hospital	1649	747	45.3	1.43	(0.92-2.27)			0.74	(0.44-1.28)		
	Nursing_home	97	12	12.4	0.25	(0.12-0.52)			0.18	(0.08-0.40)		
	Other_known	3494	1946	55.7	2.17	(1.40-3.42)			2.86	(1.70-4.87)		
	Unknown	306	210	68.6	3.77	(2.30-6.28)			3.68	(2.06-6.66)		
**Police district**	Agder	420	95	22.6	1	Ref.	1132	< 0.001	1	Ref.	831.2	< 0.001
	Asker og Bærum	52	39	75.0	9.97	(5.29-19.92)			5.77	(2.52-13.69)		
	Follo	150	94	62.7	5.70	(3.83-8.56)			3.02	(1.63-5.63)		
	Gudbrandsdal	143	8	5.6	0.21	(0.10-0.42)			0.16	(0.06-0.39)		
	Haugaland og Sunnhordland	125	109	87.2	22.62	(13.18-41.1)			30.47	(15.22-63.59)		
	Hedmark	375	114	30.4	1.49	(1.09-2.05)			1.25	(0.78-2.03)		
	Helgeland	105	70	66.7	6.77	(4.29-10.86)			5.81	(2.68-12.74)		
	Hordaland	400	344	86.0	20.78	(14.57-30.09)			17.51	(9.78-31.67)		
	Midtre Hålogaland	151	89	58.9	4.88	(3.30-7.28)			4.27	(2.45-7.48)		
	Nord-Trøndelag	167	65	38.9	2.18	(1.48-3.20)			1.62	(0.94-2.80)		
	Nordmøre og Romsdal	141	53	37.6	2.06	(1.37-3.10)			2.07	(1.16-3.68)		
	Nordre Buskerud	171	65	38.0	2.10	(1.43-3.07)			1.49	(0.84-2.64)		
	Oslo	626	355	56.7	4.46	(3.39-5.91)			4.21	(2.41-7.38)		
	Rogaland	289	251	86.9	22.27	(14.95-33.91)			18.61	(10.09-34.83)		
	Romerike	218	113	51.8	3.67	(2.59-5.22)			2.31	(1.29-4.18)		
	Salten	110	80	72.7	9.00	(5.65-14.65)			8.47	(3.82-19.2)		
	Sogn og Fjordane	152	86	56.6	4.43	(3.00-6.59)			4.64	(2.64-8.21)		
	Sunnmøre	134	27	20.1	0.87	(0.53-1.39)			0.90	(0.47-1.72)		
	Søndre Buskerud	203	113	55.7	4.27	(3.00-6.13)			2.92	(1.66-5.16)		
	Sør-Trøndelag	344	218	63.4	5.89	(4.31-8.11)			4.83	(2.77-8.50)		

	Telemark	210	148	70.5	8.10	(5.6-11.83)			9.21	(5.4-15.87)		
	Troms	243	122	50.2	3.44	(2.45-4.84)			2.85	(1.60-5.10)		
	Vestfinnmark	81	24	29.6	1.45	(0.85-2.43)			0.77	(0.37-1.60)		
	Vestfold	208	88	42.3	2.50	(1.75-3.58)			3.47	(1.98-6.13)		
	Vestoppland	159	26	16.4	0.68	(0.41-1.07)			0.67	(0.35-1.23)		
	Østfinnmark	49	18	36.7	2.00	(1.06-3.68)			0.78	(0.18-3.07)		
	Østfold	205	132	64.4	6.14	(4.28-8.89)			6.65	(3.64-12.25)		
**Municipality population**	< 2000	251	132	52.6	1	Ref.	121.9	< 0.001	1	Ref.	11.8	0.04
	2000-4999	826	417	50.5	0.92	(0.69-1.22)			0.91	(0.64-1.29)		
	5000-9999	863	397	46.0	0.77	(0.58-1.02)			0.83	(0.58-1.18)		
	10000-19999	913	513	56.2	1.16	(0.87-1.53)			1.03	(0.69-1.52)		
	20000-49999	1064	442	41.5	0.64	(0.49-0.84)			0.76	(0.51-1.13)		
	> 50000	1714	1045	61.0	1.41	(1.08-1.84)			1.15	(0.73-1.80)		
**Urbanity index**	0B (Most rural)	868	406	46.8	1	Ref.	133.8	< 0.001	1	Ref.	7.5	0.27
	0A	197	121	61.4	1.81	(1.32-2.48)			1.11	(0.72-1.72)		
	1B	254	128	50.4	1.16	(0.87-1.53)			1.04	(0.71-1.53)		
	1A	276	145	52.5	1.26	(0.96-1.65)			1.47	(0.94-2.32)		
	2B	428	198	46.3	0.98	(0.78-1.24)			1.15	(0.78-1.69)		
	2A	921	363	39.4	0.74	(0.61-0.89)			0.88	(0.57-1.34)		
	3A (Most urban)	2687	1585	59.0	1.64	(1.40-1.91)			1.08	(0.70-1.66)		
**Distance to autopsy facility (km)**	0-49	2063	1306	63.3	1	Ref.	319.5	< 0.001	1	Ref.	28	0.002
	50-99	938	518	55.2	0.71	(0.61-0.84)			0.67	(0.51-0.88)		
	100-149	920	402	43.7	0.45	(0.38-0.53)			0.56	(0.38-0.84)		
	150-199	469	266	56.7	0.76	(0.62-0.93)			0.75	(0.46-1.22)		
	200-249	312	133	42.6	0.43	(0.34-0.55)			0.51	(0.30-0.86)		
	250-299	192	39	20.3	0.15	(0.10-0.21)			0.35	(0.18-0.67)		
	300-349	309	106	34.3	0.30	(0.24-0.39)			0.72	(0.41-1.26)		
	350-399	197	60	30.5	0.26	(0.18-0.35)			0.47	(0.24-0.91)		
	400-449	90	38	42.2	0.43	(0.28-0.65)			0.94	(0.43-2.06)		
	450-499	92	58	63.0	0.98	(0.64-1.52)			0.86	(0.37-2.04)		
	> 500	49	20	40.8	0.40	(0.22-0.71)			1.69	(0.41-7.51)		

4. Accidental falls

		Deaths	Forensic autopsies	Per cent
**Total**		**20306**	**1050**	**5.2**

Variable	Level	Deaths	For.aut.	Per cent	OR (univariate)	95% CI	LRT (univariate)	p value	OR (multi predictor)	95% CI	LRT (multi predictor)	p value
**Year**	1996-2002	6199	352	5.7	1	Ref.	6.03	0.05	1	Ref.	4.78	0.09
	2003-2010	6906	357	5.2	0.91	(0.78-1.05)			0.84	(0.68-1.03)		
	2011-2017	7201	341	4.7	0.83	(0.71-0.96)			1.03	(0.84-1.27)		
**Sex**	Male	8870	774	8.7	1	Ref.	410.2	< 0.001	1	Ref.	8.93	0.002
	Female	11436	276	2.4	0.26	(0.22-0.30)			0.75	(0.63-0.91)		
**Age**	20-29	114	70	61.4	1	Ref.	3015	< 0.001	1	Ref.	1069	< 0.001
	0-9	16	6	37.5	0.39	(0.13-1.09)			0.45	(0.12-1.59)		
	10-19	50	28	56.0	0.80	(0.41-1.57)			1.04	(0.48-2.27)		
	30-39	121	71	58.7	0.89	(0.53-1.50)			0.95	(0.51-1.75)		
	40-49	231	127	55.0	0.77	(0.49-1.21)			1.04	(0.61-1.77)		
	50-59	468	220	47.0	0.56	(0.37-0.85)			0.75	(0.46-1.22)		
	60-69	813	205	25.2	0.21	(0.14-0.32)			0.34	(0.21-0.55)		
	70-79	2782	168	6.0	0.04	(0.03-0.06)			0.10	(0.06-0.16)		
	80-89	9227	128	1.4	0.01	(0.01-0.01)			0.03	(0.02-0.05)		
	90 and above	6484	27	0.4	0.003	(0-0.005)			0.01	(0.01-0.02)		
**Place of death**	At_home	1306	338	25.9	1	Ref.	2457	< 0.001	1	Ref.	872.4	< 0.001
	Hospital	9786	343	3.5	0.10	(0.09-0.12)			0.09	(0.07-0.12)		
	Nursing_home	8470	23	0.3	0.01	(0.01-0.01)			0.02	(0.01-0.04)		
	Other_known	524	254	48.5	2.69	(2.18-3.33)			1.22	(0.90-1.65)		
	Unknown	220	92	41.8	2.06	(1.53-2.76)			2.08	(1.39-3.10)		
**Police district**	Agder	1116	22	2.0	1	Ref.	386.1	< 0.001	1	Ref.	226.2	< 0.001
	Asker og Bærum	609	18	3.0	1.52	(0.81-2.83)			2.89	(0.87-9.75)		
	Follo	335	13	3.9	2.04	(1.00-3.99)			2.77	(0.78-9.99)		
	Gudbrandsdal	438	5	1.1	0.62	(0.22-1.47)			0.50	(0.12-2.09)		
	Haugaland og Sunnhordland	517	35	6.8	3.58	(2.1-6.22)			11.81	(4.09-35.44)		
	Hedmark	937	12	1.3	0.66	(0.32-1.30)			1.62	(0.52-5.15)		
	Helgeland	345	11	3.2	1.67	(0.79-3.37)			4.26	(1.01-18.04)		
	Hordaland	1614	196	12.1	6.74	(4.42-10.78)			15.21	(5.49-44.11)		
	Midtre Hålogaland	556	20	3.6	1.86	(1.01-3.42)			2.33	(0.81-6.75)		
	Nord-Trøndelag	574	20	3.5	1.80	(0.97-3.31)			4.10	(1.39-12.39)		
	Nordmøre og Romsdal	530	16	3.0	1.56	(0.81-2.96)			0.95	(0.31-2.97)		
	Nordre Buskerud	446	16	3.6	1.86	(0.96-3.54)			3.77	(1.16-12.52)		
	Oslo	2606	214	8.2	4.36	(2.87-6.96)			6.63	(2.38-19.31)		
	Rogaland	956	91	9.5	5.14	(3.28-8.42)			12.51	(4.43-36.96)		
	Romerike	1081	33	3.1	1.55	(0.91-2.70)			3.22	(1.05-10.23)		
	Salten	362	21	5.8	3.06	(1.67-5.62)			3.23	(0.73-14.7)		
	Sogn og Fjordane	466	27	5.8	3.04	(1.73-5.42)			2.06	(0.75-5.84)		
	Sunnmøre	554	8	1.4	0.76	(0.32-1.62)			0.46	(0.13-1.55)		
	Søndre Buskerud	853	22	2.6	1.32	(0.73-2.39)			2.26	(0.74-7.11)		
	Sør-Trøndelag	1203	69	5.7	2.98	(1.87-4.93)			5.30	(1.86-15.78)		

	Telemark	846	38	4.5	2.32	(1.38-3.98)			5.98	(2.18-17.01)		
	Troms	476	36	7.6	4.03	(2.37-6.99)			3.04	(1.01-9.45)		
	Vestfinnmark	143	4	2.8	1.57	(0.49-4.01)			1.55	(0.33-6.55)		
	Vestfold	976	38	3.9	2.00	(1.19-3.43)			4.99	(1.71-15.18)		
	Vestoppland	608	11	1.8	0.94	(0.44-1.88)			2.28	(0.69-7.63)		
	Østfinnmark	102	9	8.8	4.94	(2.15-10.57)			163.0	(5.94-4252.3)		
	Østfold	1057	45	4.3	2.19	(1.32-3.71)			5.17	(1.65-16.72)		
**Municipality population**	< 2000	408	29	7.1	1	Ref.	139.7	< 0.001	1	Ref.	10.14	0.07
	2000-4999	1451	78	5.4	0.74	(0.48-1.16)			1.35	(0.69-2.67)		
	5000-9999	2118	94	4.4	0.60	(0.40-0.94)			1.51	(0.77-2.99)		
	10000-19999	3069	102	3.3	0.44	(0.29-0.69)			1.25	(0.61-2.60)		
	20000-49999	5197	165	3.2	0.42	(0.29-0.65)			1.76	(0.83-3.75)		
	> 50000	8063	582	7.2	1.00	(0.69-1.50)			2.48	(1.16-5.37)		
**Urbanity index**	0B (Most rural)	1736	93	5.4	1	Ref.	85.6	< 0.001	1	Ref.	5.94	0.43
	0A	430	32	7.4	1.43	(0.94-2.15)			1.23	(0.55-2.74)		
	1B	878	33	3.8	0.70	(0.46-1.03)			1.40	(0.70-2.76)		
	1A	748	31	4.1	0.77	(0.50-1.15)			1.19	(0.51-2.74)		
	2B	1623	53	3.3	0.60	(0.42-0.84)			1.92	(0.98-3.77)		
	2A	4256	138	3.2	0.59	(0.45-0.78)			0.98	(0.46-2.09)		
	3A (Most urban)	10635	670	6.3	1.18	(0.95-1.49)			0.88	(0.40-1.94)		
**Distance to autopsy facility (km)**	0-49	9014	626	6.9	1	Ref.	122.7	< 0.001	1	Ref.	10.13	0.43
	50-99	3021	122	4.0	0.57	(0.46-0.69)			0.86	(0.52-1.41)		
	100-149	3470	120	3.5	0.48	(0.39-0.59)			0.54	(0.28-1.06)		
	150-199	1283	69	5.4	0.77	(0.59-0.98)			0.90	(0.39-2.07)		
	200-249	682	27	4.0	0.56	(0.37-0.81)			0.62	(0.24-1.63)		
	250-299	560	16	2.9	0.41	(0.24-0.64)			0.75	(0.23-2.42)		
	300-349	979	29	3.0	0.42	(0.28-0.59)			0.75	(0.26-2.19)		
	350-399	516	11	2.1	0.30	(0.16-0.52)			0.65	(0.16-2.68)		
	400-449	327	13	4.0	0.57	(0.32-0.96)			0.62	(0.15-2.49)		
	450-499	328	8	2.4	0.36	(0.16-0.66)			0.37	(0.07-1.83)		
	> 500	126	9	7.1	1.08	(0.52-2.00)			0.04	(0-1.08)		

5. Accidental poisonings

	Deaths	Forensic autopsies	Per cent
**Total**	**7719**	**6090**	**78.9**

Variable	Level	Deaths	For.aut.	Per cent	OR (univariate)	95% CI	LRT (univariate)	p value	OR (multi predictor)	95% CI	LRT (multi predictor)	p value
**Year**	1996-2002	2363	1961	83.0	1	Ref.	36.7	< 0.001	1	Ref.	7.54	0.02
	2003-2010	2984	2319	77.7	0.72	(0.62-0.82)			0.84	(0.70-1.00)		
	2011-2017	2372	1810	76.3	0.66	(0.57-0.76)			1.04	(0.86-1.26)		
**Sex**	Male	5734	4624	80.6	1	Ref.	39.5	< 0.001	1	Ref.	2.72	0.1
	Female	1985	1466	73.9	0.68	(0.60-0.76)			1.14	(0.98-1.34)		
**Age**	20-29	1551	1374	88.6	1	Ref.	694.9	< 0.001	1	Ref.	384.5	< 0.001
	0-9	4	4	100	1.16	(0.12-154.3)			1.53	(0.11-225.1)		
	10-19	142	133	93.7	1.81	(0.97-3.82)			3.73	(1.74-8.86)		
	30-39	2088	1785	85.5	0.76	(0.62-0.93)			0.66	(0.52-0.83)		
	40-49	1796	1413	78.7	0.48	(0.39-0.58)			0.44	(0.35-0.55)		
	50-59	1268	950	74.9	0.39	(0.31-0.47)			0.30	(0.24-0.38)		
	60-69	533	342	64.2	0.23	(0.18-0.29)			0.18	(0.13-0.24)		
	70-79	180	62	34.4	0.07	(0.05-0.10)			0.07	(0.05-0.11)		
	80-89	112	17	15.2	0.02	(0.01-0.04)			0.04	(0.02-0.07)		
	90 and above	45	10	22.2	0.04	(0.02-0.07)			0.09	(0.04-0.20)		
**Place of death**	At_home	3752	3107	82.8	1	Ref.	1250	< 0.001	1	Ref.	1009	< 0.001
	Hospital	1052	394	37.5	0.12	(0.11-0.14)			0.07	(0.06-0.09)		
	Nursing_home	166	83	50.0	0.21	(0.15-0.28)			0.23	(0.15-0.34)		
	Other_known	1967	1781	90.5	1.98	(1.67-2.36)			1.52	(1.25-1.85)		
	Unknown	782	725	92.7	2.62	(2.00-3.51)			2.37	(1.76-3.25)		
**Police district**	Agder	350	163	46.6	1	Ref.	962.8	< 0.001	1	Ref.	453.9	< 0.001
	Asker og Bærum	236	202	85.6	6.73	(4.48-10.35)			11.68	(4.81-28.84)		
	Follo	175	126	72.0	2.93	(1.99-4.36)			2.92	(1.25-6.89)		
	Gudbrandsdal	66	30	45.5	0.96	(0.56-1.62)			2.47	(0.93-6.65)		
	Haugaland og Sunnhordland	227	204	89.9	9.98	(6.31-16.41)			30.47	(13.54-70.31)		
	Hedmark	210	109	51.9	1.24	(0.88-1.74)			2.38	(1.19-4.78)		
	Helgeland	59	51	86.4	6.95	(3.43-15.83)			22.08	(5.79-94.9)		
	Hordaland	842	770	91.4	12.19	(8.9-16.86)			34.75	(15.56-78.53)		
	Midtre Hålogaland	82	56	68.3	2.45	(1.49-4.11)			5.83	(2.53-13.71)		
	Nord-Trøndelag	93	59	63.4	1.98	(1.24-3.19)			4.80	(2.15-10.91)		
	Nordmøre og Romsdal	104	59	56.7	1.50	(0.97-2.34)			2.96	(1.24-7.06)		
	Nordre Buskerud	109	77	70.6	2.73	(1.74-4.38)			3.87	(1.65-9.18)		
	Oslo	2141	1941	90.7	11.1	(8.61-14.35)			22.21	(10.13-49.2)		
	Rogaland	440	372	84.5	6.24	(4.49-8.74)			19.61	(8.75-44.5)		
	Romerike	299	158	52.8	1.28	(0.94-1.75)			1.95	(0.86-4.46)		
	Salten	104	80	76.9	3.77	(2.32-6.31)			20.54	(6.34-68.35)		
	Sogn og Fjordane	53	43	81.1	4.75	(2.43-10.11)			20.85	(7.05-67.32)		
	Sunnmøre	79	35	44.3	0.91	(0.56-1.49)			1.52	(0.61-3.81)		
	Søndre Buskerud	401	317	79.1	4.31	(3.14-5.95)			7.26	(3.3-16.14)		
	Sør-Trøndelag	337	274	81.3	4.96	(3.53-7.03)			11.29	(4.97-25.91)		

	Telemark	277	239	86.3	7.13	(4.83-10.76)			22.27	(10.11-49.88)		
	Troms	119	80	67.2	2.34	(1.52-3.64)			7.30	(3.04-17.72)		
	Vestfinnmark	35	16	45.7	0.97	(0.48-1.93)			2.48	(0.84-7.31)		
	Vestfold	317	198	62.5	1.90	(1.40-2.60)			4.55	(2.16-9.68)		
	Vestoppland	135	76	56.3	1.47	(0.99-2.20)			2.81	(1.29-6.16)		
	Østfinnmark	39	25	64.1	2.02	(1.04-4.06)			16.91	(2.67-129.0)		
	Østfold	390	330	84.6	6.26	(4.46-8.9)			22.27	(9.67-51.97)		
**Municipality population**	< 2000	87	54	62.1	1	Ref.	324.6	< 0.001	1	Ref.	4.58	0.47
	2000-4999	302	210	69.5	1.40	(0.85-2.29)			1.06	(0.57-1.97)		
	5000-9999	512	361	70.5	1.47	(0.91-2.34)			1.37	(0.72-2.57)		
	10000-19999	786	572	72.8	1.64	(1.03-2.58)			1.47	(0.76-2.83)		
	20000-49999	1581	1066	67.4	1.27	(0.81-1.97)			1.59	(0.82-3.02)		
	> 50000	4451	3827	86.0	3.77	(2.41-5.81)			1.67	(0.83-3.33)		
**Urbanity index**	0B (Most rural)	333	226	67.9	1	Ref.	197.5	< 0.001	1	Ref.	9.66	0.14
	0A	84	63	75.0	1.40	(0.83-2.45)			1.45	(0.69-3.12)		
	1B	129	93	72.1	1.22	(0.78-1.91)			1.20	(0.63-2.35)		
	1A	192	124	64.6	0.86	(0.59-1.26)			1.11	(0.55-2.25)		
	2B	337	226	67.1	0.96	(0.70-1.33)			1.05	(0.60-1.83)		
	2A	1311	916	69.9	1.10	(0.85-1.42)			1.01	(0.54-1.90)		
	3A (Most urban)	5333	4442	83.3	2.37	(1.85-3.00)			1.90	(0.99-3.61)		
**Distance to autopsy facility (km)**	0-49	4710	4005	85.0	1	Ref.	374.5	< 0.001	1	Ref.	9.19	0.51
	50-99	939	722	76.9	0.59	(0.49-0.70)			0.89	(0.63-1.27)		
	100-149	994	679	68.3	0.38	(0.32-0.44)			1.23	(0.76-2.00)		
	150-199	318	245	77.0	0.59	(0.45-0.78)			1.71	(0.86-3.43)		
	200-249	116	84	72.4	0.46	(0.31-0.70)			2.22	(0.99-5.04)		
	250-299	130	69	53.1	0.20	(0.14-0.28)			1.80	(0.74-4.42)		
	300-349	244	127	52.0	0.19	(0.15-0.25)			1.45	(0.65-3.24)		
	350-399	122	67	54.9	0.21	(0.15-0.31)			1.41	(0.56-3.60)		
	400-449	59	32	54.2	0.21	(0.12-0.35)			1.68	(0.58-4.87)		
	450-499	46	36	78.3	0.61	(0.32-1.29)			0.80	(0.20-3.25)		
	> 500	41	24	58.5	0.25	(0.13-0.46)			0.42	(0.06-2.61)		

6. Other external causes of death

		Deaths	Forensic autopsies	Per cent
**Total**		**9094**	**3601**	**39.6**

Variable	Level	Deaths	For.aut.	Per cent	OR (univariate)	95% CI	LRT (univariate)	p value	OR (multi predictor)	95% CI	LRT (multi predictor)	p value
**Year**	1996-2002	3028	1307	43.2	1	Ref.	24.14	< 0.001	1	Ref.	4.36	0.11
	2003-2010	3368	1280	38.0	0.81	(0.73-0.89)			0.90	(0.79-1.04)		
	2011-2017	2698	1014	37.6	0.79	(0.71-0.88)			1.05	(0.90-1.22)		
**Sex**	Male	5659	2720	48.1	1	Ref.	462.3	< 0.001	1	Ref.	0.27	0.6
	Female	3435	881	25.6	0.37	(0.34-0.41)			1.04	(0.90-1.19)		
**Age**	20-29	524	380	72.5	1	Ref.	2487	< 0.001	1	Ref.	699.1	< 0.001
	0-9	220	134	60.9	0.59	(0.42-0.82)			0.86	(0.58-1.26)		
	10-19	225	145	64.4	0.69	(0.49-0.96)			0.69	(0.47-1.01)		
	30-39	634	455	71.8	0.96	(0.74-1.25)			0.89	(0.66-1.19)		
	40-49	838	592	70.6	0.91	(0.72-1.16)			0.80	(0.61-1.06)		
	50-59	1046	669	64.0	0.67	(0.53-0.85)			0.62	(0.47-0.81)		
	60-69	1099	547	49.8	0.38	(0.30-0.47)			0.41	(0.31-0.53)		
	70-79	1276	367	28.8	0.15	(0.12-0.19)			0.20	(0.15-0.26)		
	80-89	2047	268	13.1	0.06	(0.05-0.07)			0.12	(0.09-0.16)		
	90 and above	1185	44	3.7	0.01	(0.01-0.02)			0.04	(0.03-0.07)		
**Place of death**	At_home	1603	986	61.5	1	Ref.	3603	< 0.001	1	Ref.	1713	< 0.001
	Hospital	2476	451	18.2	0.14	(0.12-0.16)			0.09	(0.07-0.10)		
	Nursing_home	1967	42	2.1	0.01	(0.01-0.02)			0.02	(0.02-0.03)		
	Other_known	2581	1746	67.6	1.31	(1.15-1.49)			0.98	(0.84-1.16)		
	Unknown	467	376	80.5	2.58	(2.02-3.32)			2.41	(1.82-3.21)		
**Police district**	Agder	557	142	25.5	1	Ref.	435.6	< 0.001	1	Ref.	349.8	< 0.001
	Asker og Bærum	187	76	40.6	2.00	(1.41-2.83)			3.91	(1.95-7.85)		
	Follo	186	75	40.3	1.97	(1.39-2.80)			2.43	(1.21-4.88)		
	Gudbrandsdal	152	21	13.8	0.48	(0.28-0.77)			0.40	(0.18-0.85)		
	Haugaland og Sunnhordland	281	169	60.1	4.39	(3.25-5.97)			6.20	(3.53-10.95)		
	Hedmark	391	88	22.5	0.85	(0.63-1.15)			0.94	(0.56-1.57)		
	Helgeland	205	88	42.9	2.20	(1.57-3.07)			2.26	(1.17-4.41)		
	Hordaland	827	441	53.3	3.33	(2.64-4.22)			7.02	(4.04-12.21)		
	Midtre Hålogaland	302	105	34.8	1.56	(1.15-2.11)			1.81	(1.09-3.02)		
	Nord-Trøndelag	273	86	31.5	1.35	(0.98-1.85)			1.70	(0.98-2.94)		
	Nordmøre og Romsdal	294	72	24.5	0.95	(0.68-1.31)			0.79	(0.46-1.37)		
	Nordre Buskerud	203	78	38.4	1.82	(1.30-2.56)			2.15	(1.18-3.95)		
	Oslo	1025	455	44.4	2.33	(1.86-2.93)			6.05	(3.46-10.63)		
	Rogaland	385	196	50.9	3.02	(2.30-3.99)			5.41	(3.00-9.80)		
	Romerike	320	98	30.6	1.29	(0.95-1.75)			1.49	(0.80-2.78)		
	Salten	188	96	51.1	3.04	(2.16-4.29)			3.65	(1.76-7.63)		
	Sogn og Fjordane	235	93	39.6	1.91	(1.38-2.64)			2.51	(1.45-4.35)		
	Sunnmøre	242	45	18.6	0.67	(0.46-0.97)			0.59	(0.32-1.06)		
	Søndre Buskerud	327	145	44.3	2.32	(1.74-3.11)			3.79	(2.08-6.92)		
	Sør-Trøndelag	531	233	43.9	2.28	(1.77-2.95)			3.91	(2.22-6.90)		

	Telemark	373	180	48.3	2.72	(2.06-3.60)			4.89	(2.88-8.34)		
	Troms	313	116	37.1	1.72	(1.28-2.32)			1.63	(0.91-2.93)		
	Vestfinnmark	130	52	40.0	1.95	(1.31-2.90)			1.48	(0.78-2.79)		
	Vestfold	446	163	36.5	1.68	(1.28-2.21)			2.04	(1.18-3.53)		
	Vestoppland	197	59	29.9	1.25	(0.87-1.79)			1.29	(0.71-2.35)		
	Østfinnmark	92	45	48.9	2.79	(1.78-4.38)			2.69	(0.97-7.56)		
	Østfold	432	184	42.6	2.16	(1.66-2.84)			3.73	(2.07-6.72)		
**Municipality population**	< 2000	392	179	45.7	1	Ref.	63.7	< 0.001	1	Ref.	9.24	0.1
	2000-4999	1154	530	45.9	1.01	(0.8-1.27)			1.18	(0.87-1.60)		
	5000-9999	1165	433	37.2	0.70	(0.56-0.89)			1.27	(0.91-1.77)		
	10000-19999	1285	532	41.4	0.84	(0.67-1.06)			1.60	(1.11-2.31)		
	20000-49999	1929	642	33.3	0.59	(0.48-0.74)			1.61	(1.10-2.36)		
	> 50000	3169	1285	40.5	0.81	(0.66-1.00)			1.49	(0.99-2.25)		
**Urbanity index**	0B (Most rural)	1316	551	41.9	1	Ref.	36.8	< 0.001	1	Ref.	4.44	0.61
	0A	283	125	44.2	1.10	(0.85-1.42)			0.91	(0.60-1.38)		
	1B	418	166	39.7	0.92	(0.73-1.14)			1.09	(0.76-1.58)		
	1A	339	133	39.2	0.90	(0.70-1.14)			1.45	(0.91-2.31)		
	2B	769	259	33.7	0.71	(0.59-0.85)			1.18	(0.85-1.66)		
	2A	1666	585	35.1	0.75	(0.65-0.87)			1.12	(0.75-1.68)		
	3A (Most urban)	4303	1782	41.4	0.98	(0.87-1.11)			1.15	(0.76-1.74)		
**Distance to autopsy facility (km)**	0-49	3543	1505	42.5	1	Ref.	96.2	< 0.001	1	Ref.	16.8	0.08
	50-99	1348	563	41.8	0.97	(0.86-1.10)			0.87	(0.64-1.17)		
	100-149	1504	558	37.1	0.80	(0.71-0.09)			0.69	(0.47-1.02)		
	150-199	695	308	44.3	1.08	(0.91-1.27)			0.92	(0.57-1.49)		
	200-249	428	175	40.9	0.94	(0.76-1.15)			0.90	(0.54-1.52)		
	250-299	297	80	26.9	0.50	(0.38-0.65)			0.81	(0.44-1.49)		
	300-349	487	146	30.0	0.58	(0.47-0.71)			0.69	(0.39-1.22)		
	350-399	317	85	26.8	0.50	(0.38-0.64)			0.57	(0.29-1.10)		
	400-449	208	66	31.7	0.63	(0.47-0.85)			0.48	(0.24-0.96)		
	450-499	163	66	40.5	0.92	(0.67-1.27)			1.07	(0.49-2.35)		
	> 500	104	49	47.1	1.21	(0.82-1.78)			0.67	(0.24-1.88)		

7. Suicides

		Deaths	Forensic autopsies	Per cent
**Total**		**11984**	**7639**	**63.7**

Variable	Level	Deaths	For.aut.	Per cent	OR (univariate)	95% CI	LRT (univariate)	p value	OR (multi predictor)	95% CI	LRT (multi predictor)	p value
**Year**	1996-2002	3769	2402	63.7	1	Ref.	74.04	< 0.001	1	Ref.	74.12	< 0.001
	2003-2010	4212	2498	59.3	0.83	(0.76-0.91)			0.70	(0.63-0.79)		
	2011-2017	4003	2739	68.4	1.23	(1.12-1.35)			1.15	(1.02-1.30)		
**Sex**	Male	8561	5278	61.7	1	Ref.	57.56	< 0.001	1	Ref.	36.62	< 0.001
	Female	3423	2361	69.0	1.38	(1.27-1.51)			1.40	(1.26-1.56)		
**Age**	20-29	2057	1444	70.2	1	Ref.	193.7	< 0.001	1	Ref.	160.4	< 0.001
	0-9	2	1	50.0	0.42	(0.02-10.75)			0.35	(0-29.65)		
	10-19	674	409	60.7	0.66	(0.55-0.79)			0.76	(0.61-0.96)		
	30-39	2061	1407	68.3	0.91	(0.80-1.04)			0.89	(0.75-1.05)		
	40-49	2291	1504	65.6	0.81	(0.71-0.92)			0.74	(0.63-0.87)		
	50-59	2084	1334	64.0	0.76	(0.66-0.86)			0.68	(0.58-0.80)		
	60-69	1395	833	59.7	0.63	(0.55-0.73)			0.56	(0.47-0.67)		
	70-79	904	468	51.8	0.46	(0.39-0.54)			0.45	(0.37-0.55)		
	80-89	456	216	47.4	0.38	(0.31-0.47)			0.27	(0.20-0.35)		
	90 and above	60	23	38.3	0.26	(0.15-0.44)			0.16	(0.08-0.32)		
**Place of death**	At_home	6145	3736	60.8	1	Ref.	336.6	< 0.001	1	Ref.	585.4	< 0.001
	Hospital	1202	601	50.0	0.64	(0.57-0.73)			0.21	(0.17-0.25)		
	Nursing_home	121	53	43.8	0.50	(0.35-0.72)			0.42	(0.26-0.69)		
	Other_known	3326	2290	68.9	1.43	(1.30-1.56)			1.52	(1.35-1.70)		
	Unknown	1190	959	80.6	2.68	(2.30-3.12)			2.74	(2.27-3.31)		
**Police district**	Agder	688	162	23.5	1	Ref.	4280	< 0.001	1	Ref.	1847	< 0.001
	Asker og Bærum	304	287	94.4	54.82	(33.55-95.48)			26.88	(13.79-54.52)		
	Follo	372	200	53.8	3.78	(2.89-4.95)			1.78	(1.08-2.95)		
	Gudbrandsdal	195	31	15.9	0.61	(0.40-0.92)			0.32	(0.17-0.61)		
	Haugaland og Sunnhordland	320	288	90.0	29.22	(19.75-44.54)			31.89	(18.7-55.51)		
	Hedmark	591	142	24.0	1.03	(0.79-1.33)			0.95	(0.62-1.47)		
	Helgeland	190	87	45.8	2.74	(1.96-3.84)			4.23	(2.37-7.59)		
	Hordaland	1032	962	93.2	44.62	(33.27-60.63)			27.62	(16.68-46.13)		
	Midtre Hålogaland	243	85	35.0	1.75	(1.27-2.40)			2.31	(1.46-3.66)		
	Nord-Trøndelag	297	91	30.6	1.43	(1.06-1.94)			0.92	(0.58-1.47)		
	Nordmøre og Romsdal	240	88	36.7	1.88	(1.37-2.58)			2.05	(1.24-3.39)		
	Nordre Buskerud	251	102	40.6	2.22	(1.63-3.02)			1.65	(1.00-2.73)		
	Oslo	1614	1508	93.4	46.19	(35.62-60.43)			25.92	(15.74-42.92)		
	Rogaland	717	656	91.5	34.92	(25.64-48.27)			22.65	(13.55-38.19)		
	Romerike	488	187	38.3	2.02	(1.57-2.60)			1.04	(0.63-1.70)		
	Salten	200	139	69.5	7.40	(5.24-10.54)			5.02	(2.64-9.62)		
	Sogn og Fjordane	190	118	62.1	5.32	(3.79-7.52)			5.14	(3.17-8.41)		
	Sunnmøre	243	43	17.7	0.70	(0.48-1.01)			0.74	(0.43-1.27)		
	Søndre Buskerud	541	375	69.3	7.33	(5.70-9.48)			3.98	(2.48-6.40)		
	Sør-Trøndelag	678	556	82.0	14.8	(11.41-19.33)			8.46	(5.22-13.8)		

	Telemark	505	412	81.6	14.38	(10.85-19.23)			14.6	(9.31-23.08)		
	Troms	301	157	52.2	3.54	(2.66-4.72)			2.08	(1.26-3.45)		
	Vestfinnmark	108	38	35.2	1.76	(1.14-2.70)			1.72	(0.93-3.18)		
	Vestfold	684	303	44.3	2.58	(2.05-3.26)			2.32	(1.48-3.66)		
	Vestoppland	324	93	28.7	1.31	(0.97-1.76)			1.21	(0.76-1.95)		
	Østfinnmark	104	53	51.0	3.37	(2.21-5.16)			4.36	(1.66-11.26)		
	Østfold	564	476	84.4	17.56	(13.23-23.53)			12.85	(7.74-21.46)		
**Municipality population**	< 2000	314	157	50.0	1	Ref.	1694	< 0.001	1	Ref.	25.71	< 0.001
	2000-4999	1218	587	48.2	0.93	(0.73-1.19)			0.85	(0.63-1.16)		
	5000-9999	1443	688	47.7	0.91	(0.71-1.16)			1.10	(0.80-1.51)		
	10000-19999	1875	965	51.5	1.06	(0.83-1.35)			1.16	(0.83-1.62)		
	20000-49999	2610	1353	51.8	1.08	(0.85-1.36)			1.36	(0.97-1.90)		
	> 50000	4524	3889	86.0	6.12	(4.83-7.76)			1.60	(1.10-2.31)		
**Urbanity index**	0B (Most rural)	1157	544	47.0	1	Ref.	905.4	< 0.001	1	Ref.	8.78	0.19
	0A	298	181	60.7	1.74	(1.35-2.26)			0.76	(0.52-1.10)		
	1B	393	194	49.4	1.10	(0.87-1.38)			1.01	(0.72-1.40)		
	1A	458	214	46.7	0.99	(0.80-1.23)			0.86	(0.58-1.28)		
	2B	804	333	41.4	0.80	(0.66-0.96)			0.73	(0.54-0.99)		
	2A	2379	1271	53.4	1.29	(1.12-1.49)			0.69	(0.49-0.96)		
	3A (Most urban)	6495	4902	75.5	3.47	(3.05-3.94)			0.72	(0.51-1.03)		
**Distance to autopsy facility (km)**	0-49	5307	4373	82.4	1	Ref.	1905	< 0.001	1	Ref.	56.85	< 0.001
	50-99	1917	1169	61.0	0.33	(0.30-0.37)			0.71	(0.57-0.87)		
	100-149	1993	967	48.5	0.20	(0.18-0.23)			0.48	(0.36-0.63)		
	150-199	760	465	61.2	0.34	(0.29-0.40)			0.50	(0.34-0.73)		
	200-249	423	167	39.5	0.14	(0.11-0.17)			0.36	(0.23-0.55)		
	250-299	308	108	35.1	0.12	(0.09-0.15)			0.50	(0.30-0.84)		
	300-349	515	142	27.6	0.08	(0.07-0.10)			0.36	(0.22-0.58)		
	350-399	331	92	27.8	0.08	(0.06-0.11)			0.40	(0.23-0.70)		
	400-449	182	57	31.3	0.10	(0.07-0.13)			0.32	(0.18-0.59)		
	450-499	141	43	30.5	0.09	(0.06-0.13)			0.15	(0.07-0.29)		
	> 500	107	56	52.3	0.23	(0.16-0.35)			0.35	(0.13-0.91)		

8. Homicides

		Deaths	Forensic autopsies	Per cent
**Total**		**874**	**844**	**96.6**

Variable	Level	Deaths	For.aut.	Per cent	OR (univariate)	95% CI	LRT (univariate)	p value	OR (multi predictor)	95% CI	LRT (multi predictor)	p value
**Year**	1996-2002	292	279	95.5	1	Ref.	2.41	0.3	1	Ref.	3.11	0.21
	2003-2010	282	276	97.9	2.05	(0.82-5.69)			2.43	(0.85-8.25)		
	2011-2017	300	289	96.3	1.22	(0.54-2.76)			1.07	(0.4-3.24)		
**Sex**	Male	512	495	96.7	1	Ref.	0.06	0.81	1	Ref.	0.36	0.55
	Female	362	349	96.4	0.91	(0.45-1.92)			0.77	(0.32-1.83)		
**Age**	20-29	162	155	95.7	1	Ref.	10.2	0.33	1	Ref.	10.2	0.33
	0-9	46	46	100	4.49	(0.53-586.2)			3.00	(0.33-462.8)		
	10-19	110	108	98.2	2.09	(0.55-11.37)			1.43	(0.33-8.44)		
	30-39	171	162	94.7	0.83	(0.30-2.20)			0.96	(0.30-2.86)		
	40-49	154	147	95.5	0.95	(0.33-2.73)			1.72	(0.50-5.95)		
	50-59	111	109	98.2	2.11	(0.55-11.47)			5.46	(0.87-54.71)		
	60-69	64	64	100	6.22	(0.74-811.9)			9.68	(0.84-1949.1)		
	70-79	32	31	96.9	1.01	(0.21-9.84)			4.75	(0.54-645.6)		
	80-89	20	18	90.0	0.36	(0.09-2.02)			1.28	(0.22-9.95)		
	90 and above	4	4	100	0.43	(0.04-59.51)			0.12	(0.01-18.38)		
**Place of death**	At_home	309	302	97.7	1	Ref.	12.1	0.02	1	Ref.	13.11	0.01
	Hospital	147	142	96.6	0.64	(0.21-2.08)			0.54	(0.14-2.09)		
	Nursing_home	4	2	50.0	0.02	(0-0.18)			0.01	(0-0.12)		
	Other_known	314	303	96.5	0.65	(0.25-1.64)			0.51	(0.17-1.39)		
	Unknown	100	95	95.0	0.43	(0.14-1.40)			0.36	(0.10-1.31)		
**Police district**	Agder	57	53	93.0	1	Ref.	40.0	0.04	1	Ref.	33.2	0.16
	Asker og Bærum	21	21	100	3.62	(0.36-487.1)			0.43	(0-896.3)		
	Follo	18	17	94.4	0.98	(0.17-10.27)			0.09	(0-102.5)		
	Gudbrandsdal	4	4	100	0.76	(0.06-106.3)			0.06	(0-525.9)		
	Haugaland og Sunnhordland	19	19	100	3.28	(0.33-442.3)			0.58	(0-560.2)		
	Hedmark	24	24	100	4.12	(0.41-554.2)			5.56	(0-10473)		
	Helgeland	10	10	100	1.77	(0.17-240.7)			0.02	(0-84.25)		
	Hordaland	67	67	100	11.36	(1.17-1517)			4.01	(0-11929)		
	Midtre Hålogaland	11	10	90.9	0.59	(0.10-6.31)			0.03	(0-57.81)		
	Nord-Trøndelag	7	7	100	1.26	(0.11-173.5)			0.48	(0-220.82)		
	Nordmøre og Romsdal	10	9	90.0	0.53	(0.08-5.74)			0.54	(0-78.35)		
	Nordre Buskerud	79	79	100	13.37	(1.38-1786)			3.59	(0-7508)		
	Oslo	216	213	98.6	5.13	(1.21-23.56)			0.68	(0-512.6)		
	Rogaland	47	45	95.7	1.53	(0.32-9.12)			0.33	(0-202.42)		
	Romerike	32	28	87.5	0.53	(0.13-2.21)			0.03	(0-33.76)		
	Salten	9	9	100	1.60	(0.15-218.3)			0.01	(0-211.7)		
	Sogn og Fjordane	8	8	100	1.43	(0.13-195.9)			0.98	(0-735.5)		
	Sunnmøre	17	17	100	2.94	(0.29-397.5)			0.40	(0-432.2)		
	Søndre Buskerud	27	27	100	4.63	(0.47-621.4)			0.26	(0-596.2)		
	Sør-Trøndelag	36	32	88.9	0.61	(0.15-2.51)			0.10	(0-68.81)		

	Telemark	35	33	94.3	1.13	(0.24-6.76)			0.14	(0-37.01)		
	Troms	25	22	88	0.54	(0.12-2.59)			0.06	(0-41.28)		
	Vestfinnmark	11	9	81.8	0.32	(0.06-2.04)			0.001	(0-2.86)		
	Vestfold	28	28	100	4.79	(0.48-643.8)			0.10	(0-217.89)		
	Vestoppland	18	18	100	3.11	(0.31-419.9)			0.09	(0-229.39)		
	Østfinnmark	11	9	81.8	0.32	(0.06-2.04)			0.001	(0-10.55)		
	Østfold	27	26	96.3	1.49	(0.26-15.37)			0.06	(0-297.82)		
**Municipality population**	< 2000	16	15	93.8	1	Ref.	7.65	0.18	1	Ref.	4.73	0.45
	2000-4999	50	50	100	9.77	(0.5-1456)			11.64	(0.12-78019)		
	5000-9999	154	149	96.8	2.63	(0.26-14.43)			0.98	(0.01-30.92)		
	10000-19999	108	100	92.6	1.14	(0.12-5.65)			0.94	(0.01-15.93)		
	20000-49999	127	122	96.1	2.16	(0.21-11.84)			2.31	(0.02-67.8)		
	> 50000	419	408	97.4	3.44	(0.36-15.97)			1.33	(0.01-25.98)		
**Urbanity index**	0B (Most rural)	56	53	94.6	1	Ref.	3.87	0.69	1	Ref.	7.38	0.28
	0A	17	16	94.1	0.72	(0.11-7.81)			0.16	(0-33.36)		
	1B	18	17	94.4	0.76	(0.12-8.26)			5.60	(0.28-1629)		
	1A	33	32	97	1.42	(0.22-15.1)			0.001	(0-2.39)		
	2B	49	47	95.9	1.24	(0.23-7.74)			0.83	(0.01-63.59)		
	2A	113	108	95.6	1.29	(0.29-5.04)			0.06	(0-5.45)		
	3A (Most urban)	588	571	97.1	2.14	(0.55-6.26)			0.11	(0-146.4)		
**Distance to autopsy facility (km)**	0-49	515	500	97.1	1	Ref.	18.5	0.05	1	Ref.	10.9	0.37
	50-99	89	87	97.8	1.08	(0.33-5.54)			5.24	(0.42-7475)		
	100-149	97	97	100	6.04	(0.8-773.3)			6.96	(0.11-4124)		
	150-199	39	36	92.3	0.32	(0.11-1.28)			0.21	(0-33.43)		
	200-249	22	20	90.9	0.25	(0.07-1.34)			0.01	(0-15.33)		
	250-299	18	17	94.4	0.36	(0.08-3.41)			0.16	(0-715.1)		
	300-349	38	35	92.1	0.31	(0.10-1.25)			0.23	(0-172.1)		
	350-399	12	11	91.7	0.24	(0.05-2.28)			0.07	(0-126.7)		
	400-449	22	21	95.5	0.44	(0.10-4.16)			0.43	(0-1540)		
	450-499	9	9	100	0.59	(0.07-77.1)			0.44	(0-7725)		
	> 500	13	11	84.6	0.14	(0.04-0.78)			2.34	(0-555802)		
